# Quorum Sensing in Emulsion Droplet Swarms Driven by a Surfactant Competition System

**DOI:** 10.1002/advs.202307919

**Published:** 2024-06-17

**Authors:** Pieter J. de Visser, Dimitrios Karagrigoriou, Anne‐Déborah C. Nguindjel, Peter A. Korevaar

**Affiliations:** ^1^ Institute for Molecules and Materials Radboud University Heyendaalseweg 135 Nijmegen 6525 AJ The Netherlands

**Keywords:** active droplets, Marangoni effect, self‐assembly, surfactants, systems chemistry

## Abstract

Quorum sensing enables unicellular organisms to probe their population density and perform behavior that exclusively occurs above a critical density. Quorum sensing is established in emulsion droplet swarms that float at a water surface and cluster above a critical density. The design involves competition between 1) a surface tension gradient that is generated upon release of a surfactant from the oil droplets, and thereby drives their mutual repulsion, and 2) the release of a surfactant precursor from the droplets, that forms a strong imine surfactant which suppresses the surface tension gradient and thereby causes droplet clustering upon capillary (Cheerios) attraction. The production of the imine‐surfactant depends on the population density of the droplets releasing the precursor so that the clustering only occurs above a critical population density. The pH‐dependence of the imine‐surfactant formation is exploited to trigger quorum sensing upon a base stimulus: dynamic droplet swarms are generated that cluster and spread upon spatiotemporally varying acid and base conditions. Next, the clustering of two droplet subpopulations is coupled to a chemical reaction that generates a fluorescent signal. It is foreseen that quorum sensing enables control mechanisms in droplet‐based systems that display collective responses in contexts of, e.g., sensing, optics, or dynamically controlled droplet‐reactors.

## Graphical abstract

The concept of quorum sensing – known to orchestrate collective behavior in unicellular organisms – is established in emulsion droplet systems. Initial repulsion between droplets upon surfactant release is counteracted when the droplets release sufficient surfactant precursor that suppresses the repulsive surface tension gradients, providing droplet clustering above a critical droplet density.

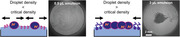

## 1 Introduction

Collective behavior provides unicellular organisms with emerging functions that are essential to survive as a multicellular community. Typically, unicellular organisms regulate collective behavior by quorum sensing: a communication mechanism by which cells sense their population density and cooperatively adapt to their environment. To employ quorum sensing, unicellular organisms excrete signaling molecules that signal their presence to neighboring peers.^[^ ^1^ ^]^ When sensing a concentration of signaling molecules that exceeds a threshold value, which implies that the population density exceeds a critical density, the community changes metabolism, motion, or structure.^[^ ^2^ ^]^ For example, colonies of the slime mold *Dictyostelium discoideum* cluster together in case of a nutrient shortage and collectively move to a more hospitable environment to form a multicellular, spore‐producing fruiting body.^[^ ^3^ ^]^ These slime molds dynamically generate concentration gradients of cyclic adenosine monophosphate (cAMP), which is a chemoattractant signaling molecule, so that upon sensing a critical concentration of cAMP, the slime molds migrate toward each other and form a cluster.^[^ ^4^ ^]^ Furthermore, colonies of *Vibrio* (or *Aliivibrio*) *fischeri* bacteria form a bioluminescent biofilm when they reach a critical population density and thereby provide camouflage to their symbiotic host, the Hawaiian bobtail squid.^[^ ^5^ ^]^

Implementing quorum sensing in synthetic chemical systems would allow for functional structures that organize from elementary units and collectively adapt to changing environments.^[^ ^6^, ^7^, ^8^, ^9^, ^10^ ^]^ To this end, quorum sensing can be understood from a chemical perspective as a system in which signaling molecules are transferred between particles, the elementary units, and upon reception lead to a macroscale physical or chemical response of the particles.^[^ ^11^, ^12^, ^13^, ^14^, ^15^ ^]^ The use of droplets, for example, oil‐in‐water emulsion droplets, as the elementary units provides an attractive strategy because they can easily be moved and their chemical composition can be controlled and manipulated.

Emulsion droplet behavior can be manipulated via chemical reactions that exclusively take place inside or outside the droplets, e.g., the formation of surfactants or compounds that are insoluble in either the droplet phase or the bulk phase. Changes in the droplet composition can lead to the mobility of droplets due to Marangoni flow or the “Cheerios effect”.^[^ ^16^, ^17^, ^18^, ^19^ ^]^ Marangoni flow is driven by a surface tension gradient that is caused by a concentration gradient of a surface‐active compound (surfactant) along an interface.^[^ ^20^, ^21^, ^22^ ^]^ This flow is directed from low surface tension regions (high surfactant concentration) toward high surface tension regions (low surfactant concentration). The Cheerios effect is the tendency of floating objects with equally shaped menisci (convex–convex or concave–concave) to aggregate, driven by capillary forces.^[^ ^23^ ^]^

Droplet‐based systems are applied and studied in an expanding range of contexts that vary from sensing,^[^ ^24^, ^25^ ^]^ reconfigurable objects,^[^ ^26^, ^27^ ^]^ optics,^[^ ^28^ ^]^ maze solving,^[^ ^29^ ^]^ chemotaxis,^[^ ^30^ ^]^ predator‐prey behavior,^[^ ^31^ ^]^ origin‐of‐life,^[^ ^32^ ^]^ self‐organization,^[^ ^33^, ^34^, ^35^, ^36^, ^37^ ^]^ and (multiphase) controlled reactors.^[^ ^38^, ^39^ ^]^ Many of these systems exploit dynamic, out‐of‐equilibrium behavior of individual droplets, rather than functions that emerge from the interactions amongst multiple droplets in a swarm with properties that cannot be established by individual droplets. The mechanisms that enable unicellular organisms to cooperate as a collective system inspired us to design a quorum‐sensing system that allows droplet swarms to collectively cluster beyond a critical population density.

**Figure 1:**
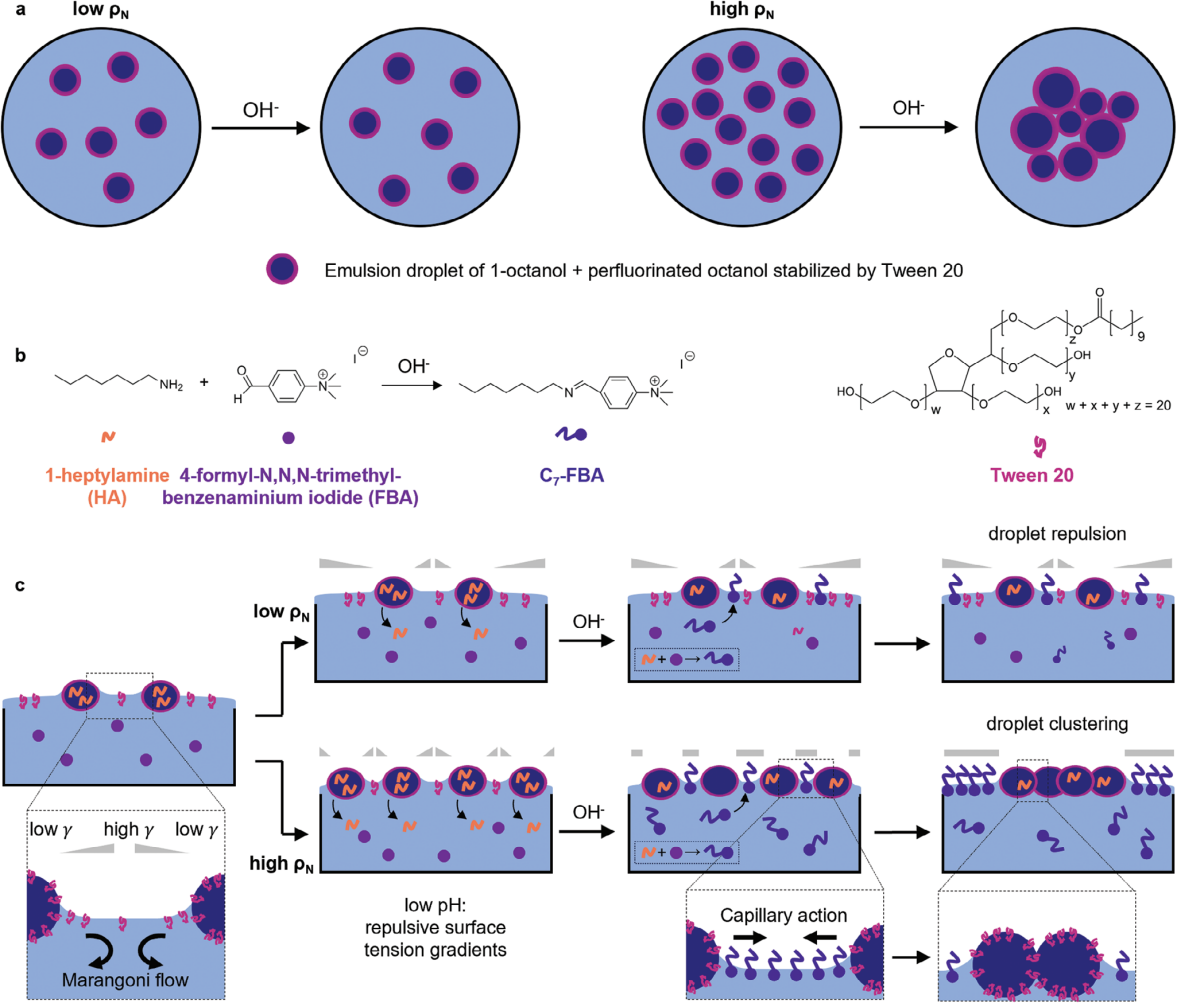
Bottom‐up design of quorum sensing droplets. a) At low droplet population density *ρ*_N_, the droplets stay separated after addition of base, whereas at high *ρ*_N_, the droplets cluster together. b) The chemical reaction that permits quorum sensing is the formation of the imine surfactant C_7_‐FBA upon the addition of base to the droplet system. This surfactant competitively adsorbs at the air–water interface with the emulsion‐stabilizing surfactant Tween 20. c) The emulsion droplets float on top of an aqueous FBA solution and repel each other due to outward Marangoni flows that are generated upon release of Tween 20 to the air–water interface. At low pH, the signaling molecule 1‐heptylamine contained in the droplets leaks into the aqueous phase as a surfactant precursor. Upon addition of base, C_7_‐FBA is formed in the aqueous phase and occupies the air–water interface. As C_7_‐FBA is a strong surfactant, it reduces the surface tension (*γ*) and thereby cancels the outward Marangoni flows due to the Tween 20 release. Therefore, the droplets cluster upon capillary (Cheerios) attraction. However, this clustering only occurs when enough droplets are present that leak 1‐heptylamine, i.e., when *ρ*_N_ is higher than a critical population density.

The bottom‐up design of our system (**Figure** **1**) comprises 1) oil‐in‐water emulsion droplets that float on water, as compartmentalized analogs to unicellular organisms; 2) surfactant molecules (Tween 20) that are released from these droplets to the air–water interface, and thereby generate outward Marangoni flow locally around the droplets, causing the droplets to repel each other, and 3) an amine surfactant precursor that is released as signaling molecule from the droplets. First, on an aqueous solution, we disperse floating emulsion droplets which initially spread out due to repulsive surface tension gradients. The amine surfactant precursor leaks from the droplets to the solution at low pH. After the addition of a base stimulus, at high pH, the amine reacts with an aldehyde in the aqueous solution to form an amphiphile via a dynamic covalent imine bond.^[^ ^40^, ^41^, ^42^ ^]^ Subsequently, this imine amphiphile adsorbs to the air–water interface, and we rationalize how it is a stronger surfactant that competes with the Tween 20 surfactant that was adsorbed to the air–water interface prior to the imine formation. Therefore, the imine amphiphile cancels the surface tension gradients that cause the initial droplet–droplet repulsion, such that the capillary (Cheerios) attraction starts to dominate and the droplets cluster. Importantly, a critical amount of imine amphiphile is required to suppress the repulsive Marangoni flow amongst the droplets. Therefore, only beyond a critical population density of droplets that release the amine surfactant precursor, the clustering response will occur – which is a quorum‐sensing response to the base stimulus. Furthermore, we show that the emulsion swarms respond to temporal fluctuations by clustering and spreading in pH cycles, and form separate dynamic clusters in spatial pH gradients. Finally, we exploit our system to create an emulsion that generates a fluorescent response upon clustering, reminiscent of the quorum‐sensing behavior of *Vibrio fischeri*.

## 2 Results and Discussion

### 2.1 pH‐Dependent Behavior of Droplet Swarms

**Figure 2:**
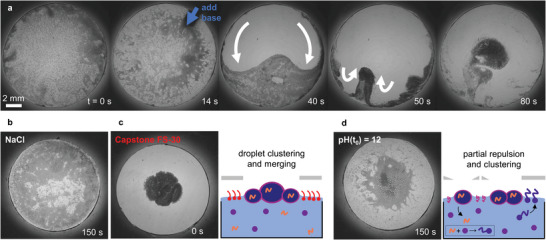
Clustering of emulsion droplets upon addition of base stimulus. Optical microscopy images of a hydrophobic PDMS well (diameter 12 mm, depth 700 µm) with floating emulsion droplets (4 µL, 400 mm HA) on an aqueous solution (120 µL, 40 mm FBA). a) Initially, the solution is acidic (pH 2) and the emulsion droplets spread over the surface of the solution. After the addition of base (7 µL, 2 m NaOH) at t = 14 s, the solution becomes alkaline (pH 12). The dark spot in the top right is the pipette tip, indicated with the blue arrow. The emulsion droplets move away from the spot where the base was added. At the opposite side of the well, the emulsion droplets cluster. Subsequently, the droplets propagate toward the center of the well, where they stay. The white arrows indicate the movement of the droplets over the air–water interface. b) Adding NaCl (7 µL, 2 m) instead of NaOH does not lead to droplet clustering, which means that convection or the increase in ionic strength is not a driving force for droplet clustering. c) The emulsion droplets immediately cluster and merge at pH 2 when a strong surfactant is included in the aqueous solution (Capstone FS‐30, 0.116 wt.%), which suppresses the repulsive Marangoni flow between droplets due to the release of Tween 20. d) The droplets cluster less tightly when the aqueous solution is initially basic as lower amounts of 1‐heptylamine leak from the droplets into the aqueous solution.

The oil‐in‐water droplets are comprised of an organic phase of 1‐octanol and perfluorinated octanol (80:20 v/v%) and are loaded with 1‐heptylamine (HA) as the signaling molecule. We emulsify 10 v% of this organic phase in an aqueous solution of the surfactant polyoxyethylene (20) sorbitan monolaurate, known as Tween 20 (10 mm). We selected 1‐octanol for its high boiling point, to avoid evaporation of the droplets during the experiments, and its capacity to form stable emulsions with Tween 20 while at the same time showing suitable partition of HA with water (vide infra). Furthermore, we included perfluorinated octanol in the droplets to increase their density up to ≈1 g mL^−1^, which was observed to contribute to the spreading of the droplet populations over the concave air–water interface in the experiments. At the start of a quorum sensing experiment, we disperse emulsion droplets onto an aqueous solution of 4‐formyl‐N,N,N‐trimethylbenzenaminium iodide (FBA; Figure S1, Supporting Information) at pH 2 in a thin solvophobized PDMS well to create a “droplet swarm.” Initially, the droplets spread on top of the aqueous solution, as shown in **Figure** **2**a and Video S1, Supporting Information. The droplets are roughly between 10 and 100 µm in diameter. As a base stimulus, we add an aliquot of NaOH solution (7 µL; 2 m) to the PDMS well, which increases the pH to 12. Immediately, the droplets move away from the area where the base was added due to the instantaneous surface tension gradient established by the imine surfactant C_7_‐FBA formed at high pH (Figure 2c). For emulsions with droplets that are loaded with sufficient 1‐heptylamine, the droplets subsequently cluster in the center of the well over a time course of 15–60 s after the base stimulus. Importantly, the droplet clustering is not due to trivial convection upon addition of the aliquot, as indicated by the absence of droplet movement when the same amount of water (Figure S2 and Video S2, Supporting Information) or NaCl solution (Figure 2b; Video S2, Supporting Information) was added.

The initial spreading of the floating droplets at low pH implies a repulsive force that counteracts their mutual attraction due to the Cheerios effect. We hypothesize that this spreading is driven by the partial release of the surfactant Tween 20, included to stabilize the emulsion droplets, from the droplet interface to the pristine air–water interface. This surfactant partitioning leads to local surfactant concentration gradients around the emulsion droplets, and the corresponding surface tension gradients give rise to droplet‐droplet Marangoni repulsion – analogous to the mutual repulsion of floating camphor crystals as reported by Soh et al.^[^ ^43^ ^]^ Indeed, when dispersed on an aqueous solution with a stronger surfactant, Capstone FS‐30 (0.116 wt.%, ST = 20.0 ± 0.2 mN m^−1^), the droplets were observed to cluster immediately, corroborating the role of local surface tension gradients along the air–water interface as driving force for their initial spreading (Figure 2c; Figure S2, Video S2, Supporting Information). Moreover, when the air–water interface is covered with a glass cover slide, the droplets do not move upon a base stimulus as the surface tension effects are now omitted from the system (Figure S3, Supporting Information). We note that perfluorinated octanol has no special role in the spreading and clustering mechanism: comparable results were obtained when perfluorinated octanol was substituted for diethyl phthalate as a high‐density compound to increase the density of the emulsion droplets (Figure S3, Supporting Information).

The subsequent clustering of the emulsion droplets upon the addition of base is driven by the reaction of 1‐heptylamine and FBA into the strong imine surfactant C_7_‐FBA, which competitively adsorbs at the air–water interface with Tween 20 (Figure 1). However, due to the time delay between amine release from the droplets (upon droplet deposition) and production of imine surfactant (upon base stimulus), the imine surfactant adsorbs to the air–water interface “globally,” i.e., throughout the entire well, not only locally around the droplets. Hence, the imine surfactant does not increase the local surface tension gradients established by Tween 20, but – instead – cancels these local surface tension gradients.

The imine surfactant only forms at high pH, as confirmed by ^1^H‐NMR experiments (Figure S4, Supporting Information). Even though FBA is partly present in the hydrate form (at pH 7 17%, Figure S1, Supporting Information), this is not expected to affect the formation of the imine surfactant at pH 12. Alternatively, FBA forms an acetal with octanol at pH 2, at the interface of the emulsion droplets. Earlier it has been shown how such acetal formation could lead to droplet deformation.^[^ ^44^ ^]^ Even though at pH 2 more droplet merging occurred after 1 h in the presence of FBA, compared to emulsion droplets dispersed on an aqueous solution without FBA, no significant merging of droplets was observed over a time course of 10 min – the typical timescale of our experiments (Figure S3, Supporting Information).

### 2.2 Surface Tension Measurements of Competing Surfactants

**Figure 3:**
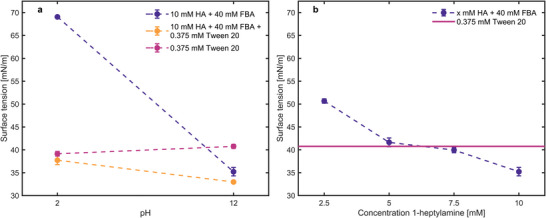
Surface tension measurements of the precursors of C_7_‐FBA, 1‐heptylamine (HA) and FBA, and Tween 20 in water. a) Surface tension at pH 2 and pH 12. b) Surface tension with varying concentrations of signaling molecule 1‐heptylamine at pH 12. For all experiments, *n* = 3, and the error bars indicate the standard deviation.

To assess the role of surfactant competition in droplet clustering, we measured the surface tension of a Tween 20 and a C_7_‐FBA solution, at concentrations representative of our experimental conditions. As shown in **Figure** **3**a, at pH 2, Tween 20 is active as a surfactant, whereas 1‐heptylamine and FBA hardly decrease the surface tension. The imine surfactant C_7_‐FBA is only formed from its precursors 1‐heptylamine and FBA under basic conditions (at pH 12) and lowers the surface tension to 35.2 mN m^−1^. In comparison, Tween 20 is a weaker surfactant as its surface tension is significantly higher (40.8 mN m^−1^). The combination of Tween 20 and C_7_‐FBA at pH 12 results in a slightly reduced surface tension of 33.0 mN m^−1^, which we ascribe to slight synergistic adsorption of both surfactants to the air–water interface. Together, these surface tension measurements show that in an aqueous solution with representative concentrations of Tween 20, 1‐heptylamine, and FBA, C_7_‐FBA is formed at high pH, which leads to a surface tension reduction of ≈8 mN m^−1^ over the surface tension of Tween 20. As C_7_‐FBA is a stronger surfactant than Tween 20, we anticipate that it dominates the surfactant monolayer at the air–water interface, thereby canceling the local, repulsive surface tension gradients generated by Tween 20 at high pH, after the base stimulus (Figure 1c). With the removal of this Marangoni repulsion, the emulsion droplets would then become mutually attractive due to the Cheerios effect.

### 2.3 Classifying Release of Signaling Molecule

To estimate the concentration of 1‐heptylamine that is required for sufficient C_7_‐FBA formation to drive the emulsion clustering, we performed surface tension measurements at various concentrations of 1‐heptylamine dissolved with 40 mm FBA in water at pH 12 (Figure 3b). Assuming the droplets only cluster if the amount of C_7_‐FBA formed reduces the surface tension below the surface tension of Tween 20 (40.8 mN m^−1^), we find that ≥7.5 mm 1‐heptylamine is required to leak from the emulsion droplets into the aqueous solution. ^1^H‐NMR experiments show that upon thorough mixing of water and the organic 1‐octanol/perfluorinated octanol/1‐heptylamine solution, the distribution coefficient for 1‐heptylamine (*K* = [HA]_organic_/[HA]_aqueous_) equals *K* = 524 at high pH, and *K* = 19.5 at low pH (Figure S5 and Table S1, Supporting Information). In a typical quorum sensing experiment with emulsion droplets that are loaded with 400 mm HA, *K* = 524 at pH 12 implies that the maximum 1‐heptylamine concentration that can be obtained in the surrounding aqueous phase equals 0.75 mm, such that the minimum concentration of 7.5 mm is not surpassed. At low pH, however, 1‐heptylamine is more protonated and partitions more to the aqueous phase, resulting in a maximum concentration of 20.5 mm with *K* = 19.5. Hence, starting at a low pH is required to enhance 1‐heptylamine leakage from the droplets to the aqueous phase, to “prepare” a solution for clustering of the emulsion droplets – as corroborated by the absence of stable clustering when starting with an aqueous solution at high pH (Figure 2d; Figure S2, Video S2, Supporting Information). Together, these results show that the formation of the C_7_‐FBA surfactant, after the base stimulus, can suppress the repulsive surface tension gradients between emulsion droplets generated by Tween 20, but only if sufficient signaling molecules (1‐heptylamine) have been released from the droplets into the aqueous solution.

### 2.4 Quantifying Droplet Clustering Dynamics

We anticipate the droplets to only cluster if sufficient C_7_‐FBA is produced. To control the formation of C_7_‐FBA, and thereby the clustering of the droplets, the design of our system provides two parameters that can be manipulated experimentally: the concentration of 1‐heptylamine in the emulsion droplets and the total volume of emulsion droplets. These two parameters are analogous to the concentration of signaling molecules excreted by cells and the population density of cells, respectively. To quantify the extent of droplet clustering in the quorum sensing experiments, we analyze the video recordings of droplet swarms before and after the addition of base.

**Figure 4:**
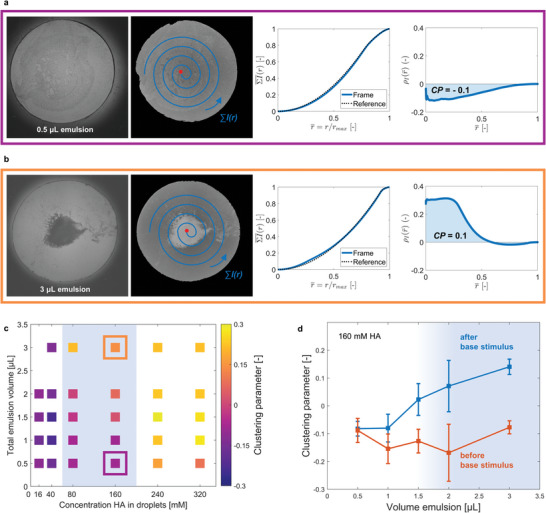
Quantification of droplet quorum sensing behavior. The image analysis is demonstrated with an individual frame for a nonclustering droplet swarm a) and a clustering droplet swarm b). a,b) First, the PDMS visible in the original frame (far left) is masked from the image, and the color scale is inverted. Next, the center of mass of the emulsion droplets is calculated (red circle), and the pixel intensities *I* are accumulated in a “spiraling walk” with increasing distance *r* away from the center of mass (left). This cumulative intensity ∑*I*(*r*) is normalized and compared to the cumulative intensity of a homogeneous reference distribution (right). Subsequently, the number of accumulated pixels is accounted for, resulting in the pixel density function *ρ*_I_($\bar{r}$) (far right). Finally, the clustering parameter *CP* is calculated by taking the integral of the pixel density function. For a nonclustering droplet swarm *CP* < 0, whereas *CP* > 0 for a clustering droplet swarm. c) Clustering phase diagram for various droplet populations, with different droplet population densities (controlled via the total emulsion volume) and loaded with different concentrations of 1‐heptylamine (HA). The purple and orange boxed data points in the phase diagram correspond to the experiments displayed in a) and b), respectively. d) Droplets loaded with 160 mm 1‐heptylamine display quorum sensing: only if sufficient droplets are present (≥2 µL, blue shaded area), the droplets cluster after application of the base stimulus. For all experiments in c), *n* = 2, except for the experiments with 160 mm 1‐heptylamine (*n* = 3). The error bars in d) indicate the standard deviation of *CP* (*n* = 3).

We quantify the extent of droplet clustering in each experiment with a clustering parameter (*CP*) using image analysis software, see Experimental Section. To determine *CP*, we first determine the center of mass of the emulsion droplets in the well (**Figure** **4**a,b). Next, we calculate the cumulative pixel intensity Σ*I* by summing up the pixel intensities as a function of the distance *r* away from the center of mass. A visual analogy for this analysis is a “spiraling walk”: a walk over all pixels starting from the center of mass and spiraling outward to pixels with increasing distance *r*, while accumulating their intensities. The resulting Σ*I* versus *r* plots are normalized to compare between experiments. To differentiate between clustered and nonclustered droplet swarms, we compute the difference between the normalized cumulative pixel intensity ($\bar{\Sigma I}$) of the experimental data and that of an entirely homogeneous reference field, which gives Δ$\bar{\Sigma I}\left(\bar{r}\right)$ versus $\bar{r}$. Subsequently, we calculate a measure for the droplet density function *ρ_I_* ($\bar{r}$) through division of Δ$\bar{\Sigma I}\left(\bar{r}\right)$ by the corresponding cumulative number of pixels versus $\bar{r}$. Finally, we obtain *CP* by integrating *ρ_I_* ($\bar{r}$) over $\bar{r}$.

Essentially, *CP* amounts to a weighted average of the change in pixel intensity with respect to the entirely homogeneous reference, which is determined such that fewer pixels at a small radius, close to the center of mass, have equal weight to a larger amount of pixels at a large radius. In this analysis, *CP* = 0 implies an entirely homogeneous spreading of droplets around their center of mass. In our experiments, *CP* varies between 0.3 and −0.3; with *CP* > 0 corresponding to clustered droplet populations, whereas a *CP* < 0 implies that the droplets are more abundant toward the edge of the PDMS well.

In the phase diagram in Figure 4c, we map the clustering parameter for various amounts of droplets (total emulsion volume: 0.5–3 µL, corresponding to ≈1000–6000 droplets) and various concentrations of 1‐heptylamine loaded in the droplets (Video S3, Supporting Information). Intriguingly, only in a narrow concentration range of 80–160 mm 1‐heptylamine in the droplets, a quorum sensing response is obtained: the clustering parameter is dependent on the total amount of droplets, and clustering only occurs if the population density is high enough (Figure 4c; Video S4, Supporting Information). For droplets with ≤40 mm HA, an emulsion volume of 3 µL is insufficient to obtain clustering, whereas a similar total 1‐heptylamine content in the system with 0.5 µL of ≥240 mm 1‐heptylamine droplets results in clustering. We ascribe this difference to the role of the concentration of Tween 20, which drives the droplet repulsion and is much higher for a 3 µL emulsion with 40 mm 1‐heptylamine than for a 0.5 µL emulsion with 240 mm HA. Thereby, the narrow 1‐heptylamine concentration window (80–160 mm) corroborates how the quorum sensing response relies on the balance between Tween 20, which drives the droplet repulsion and increases with the droplet population density, and surfactant precursor HA, which is required for the droplet attraction and increases with both the droplet population density as well as their 1‐heptylamine content.

### 2.5 Droplet Swarm Clustering Reversibility

**Figure 5:**
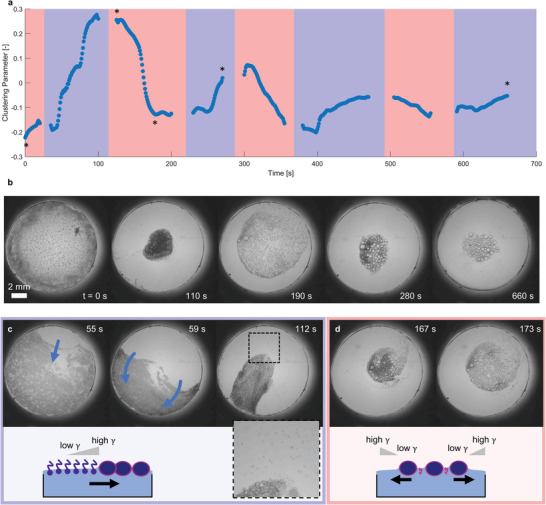
Dynamic clustering and spreading of the emulsion population upon varying base and acid conditions. a) Clustering parameter versus time, under consecutive acidic conditions (shaded in pink, pH 2) upon the addition of 7 µL 2 m HCl, and basic conditions (shaded in blue, pH 12) upon addition of 7 µL 2 m NaOH to the emulsion population (4 µL, 400 mm 1‐heptylamine). b) Microscopy images of the clustering and spreading droplets, acquired at time points marked with an asterisk (*) in (a). c) Microscopy images highlighting the spreading wave of clustering droplets, starting from the position where the base was added and indicated by the blue arrow. The scheme shows the gradient in surface tension, due to the rapid formation of the imine surfactant, leading to the droplet clustering. The inset in the bottom right shows the individual droplets that are not clustered. d) Microscopy images showing the expansion of the droplet cluster upon addition of base. The scheme shows the surface tension gradient due to the presence of Tween 20 amongst the droplets, and the lower concentration of Tween 20 in the periphery of the well as the driving force for the droplet spreading.

The pH‐sensitive imine bond allows the C_7_‐FBA amphiphile to be formed at high pH and broken at low pH. Therefore, we reasoned that the droplet clustering is susceptible to spatiotemporal pH variation. To introduce temporal pH variation, we changed the pH multiple times by adding aliquots of NaOH (7 µL, 2 m) and HCl (7 µL, 2 m) consecutively to the PDMS well. As shown in **Figure** **5**b and Video S5 (Supporting Information), the droplets indeed respond by repeatedly clustering at pH 12 and spreading at pH 2. In the first cycle, the droplets densely cluster after base addition, indicated by the large increase in the clustering parameter (Figure 5a). For the consecutive cycles, we observe that the contraction (base added) and expansion (acid added) of the droplet population occur at comparable rates. However, over time, the droplets start to merge, such that the clustering of the droplets is less pronounced and a lower rise in the clustering parameter is obtained in the consecutive cycles. We note that upon the addition of base and acid, the droplets respectively cluster and separate collectively, and no differences in repulsion or attraction amongst large and small droplets are observed.

The capillary length *L*_c_, which defines the length scale over which the capillary interactions occur, equals $\sqrt{\gamma /(\rho g)}$. In our system, with surface tension *γ* = 33.0 mN m^–1^, density of the aqueous phase *ρ* = 1000 kg m^−3^ and *g* = 9.81 m s^−2^, *L*_c_ is ≈1.8 mm. Comparable length scales have been reported in the literature for the capillary interactions amongst clustering particles at oil/water interfaces with comparable interfacial tensions, with capillary forces in the order of *F*_cap_ = 1–10 nN.^[^ ^45^ ^]^ In our system, an external force contributes to the clustering of the droplets, in addition to the capillary forces. We observe that each cycle of base addition generates a front of clustering droplets that progresses over the air–water interface, starting from the place where the base was added (Figure 5c). In cycle 1, the front initially moves with a velocity of *v* = 2 mm s^−1^, and we estimated the surface tension difference involved at ≈0.06 mN m^−1^ (Section S1, Supporting Information). This is much smaller than the maximum surface tension difference of 8 mN m^−1^ between C_7_‐FBA and Tween 20 at pH 12 (Figure 3a), which implies that the droplets start to move and cluster as soon as C_7_‐FBA starts to form upon the addition of base. After the completion of C_7_‐FBA surfactant occupation over the entire air–water interface, the droplets are anticipated to be kept together due to the capillary (Cheerios) attraction. We note that a small fraction of droplets that were left behind in the C_7_‐FBA‐rich area remained separated from the cluster and did not merge with each other, corroborating the requirement of the transient surface tension gradient to drive the rapid clustering.

Upon addition of acid, the droplets spread again, with a velocity of *v* = 170 µm s^−1^ (Figure 5d). As C_7_‐FBA hydrolyses, it can be replaced rapidly by Tween 20 amongst the clustered droplets. However, in the periphery of the well, the surface tension is transiently raised as Tween 20 needs time to reach those areas. The surface tension gradient between the cluster and the surrounding air–water interface counteracts the capillary forces amongst the droplets, and thereby drives its expansion. The forces required to separate the droplets can be approximated by *F*_Mar_ = $\frac{\partial \gamma }{\partial x}$ ∙*d* ^2^, where $\frac{\partial \gamma }{\partial x}$ equals the surface tension gradient and *d* the diameter of the droplet (Section S1, Supporting Information). For example, overcoming a capillary force of *F*_cap_ = 10 nN for a droplet of 200 µm implies Δ*γ* = 2.5 mN m^−1^ over a distance of 1 cm.

### 2.6 Droplet Swarm Clustering in Continuous pH Gradient

*Dictyostelium discoideum* populations direct their collective migration by generating localized gradients of chemoattractants. To investigate how our system responds to a spatial stimulus, we apply a pH gradient to the droplet population. We designed a PDMS well with two inlet channels that allow for the injection of acid (HCl, 2 m) and base (NaOH, 2 m) at opposite sides of the well, thereby giving rise to a pH gradient. To keep the volume of the solution in the well constant, a third channel, perpendicular to the two inlets, functions as the outlet. Automated syringe pumps regulate the injection and outflow at a constant rate so that the residence time is ≈50 min. We study the droplet dynamics by recording videos of the droplet swarms and performing Particle Image Velocimetry (PIV) on the recorded frames.^[^ ^46^ ^]^

**Figure 6:**
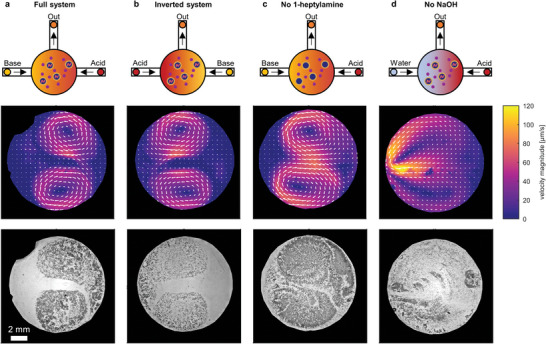
Spatiotemporal droplet population dynamics in acid‐base gradients in an aqueous FBA solution (40 mm) in a PDMS flow chamber. The top row outlines the configuration of the acid and base inlets; the middle row shows the flow profiles in the well (acquired via PIV analysis) and the bottom row shows optical microscopy images of the droplet swarms. In a) and b), the droplets are loaded with 400 mm 1‐heptylamine (2 µL), and the chamber is fed with base (2 m NaOH) and acid (2 m HCl). In c), we selected the same acid and base inlets and omitted 1‐heptylamine from the droplets; in d) we used 400 mm 1‐heptylamine droplets (2 µL) and replaced the base inlet with pure water.

When base and acid are injected into the well from opposite sides, the droplets move in concert in two oppositely rotating vortices (**Figure** **6**a,b; Video S6, Supporting Information). Irrespective of the direction of the pH gradient, these “dipolar vortices” are separated by a droplet‐free zone and rotate away from the base inlet. Intriguingly, dipolar vortices also emerge in the absence of 1‐heptylamine in the droplets (Figure 6c; Video S6, Supporting Information) or FBA in the aqueous solution (Figure S6, Video S7, Supporting Information). These observations rule out Marangoni flow resulting from C_7_‐FBA gradients as the cause of these vortices. In a control experiment where water was injected from both sides, we obtained different flow patterns that are reminiscent of “quadrupolar vortices” (Figure S6, Supporting Information). Therefore, we hypothesize that the emergence of the dipolar vortices is due to slight density differences between the injected acid (*ρ* = 1.03 g mL^−1^) and base (*ρ* = 1.08 g mL^−1^) solutions that lead to hydrodynamic instabilities via solutal buoyancy convection.^[^ ^47^ ^]^

The dynamic behavior of the droplets has been summarized in **Table** **1**. The emulsion droplets are observed to follow the convection patterns under circumstances where C_7_‐FBA is formed (Figure 6a,b) as well as under circumstances where C_7_‐FBA cannot be formed due to the absence of 1‐heptylamine (Figure 6c) or base (Figure 6d). However, strikingly, only if C_7_‐FBA is produced, the droplets cluster at the base side, and the surface tension gradient that arises due to the pH dependence of C_7_‐FBA draws the cluster toward the acid side (Figure 6a,b). Next, the cluster dissociates in part at the acid side. Together, our results show that the system responds to a nonhomogeneous application of the clustering stimulus (i.e., base) by initially moving the droplets away from the location where the clustering stimulus is applied – as was observed in Figure 2a and Figure 5c as well. We note that this concept can be used as a design principle: rather than droplets that locally reach quorum and then attract other droplets, the surface tension gradients enable droplets that have locally reached their quorum to move along a gradient and collect even more droplets to join the cluster.

**Table 1:** Overview of the dynamic behavior of droplets under varying conditions in PDMS flow chamber.

NaOH inflow	HCl inflow	1‐heptylamine in droplets	FBA in aqueous solution	Dynamic behavior
X	X	X	X	Dipolar vortices, with droplets clustering at the base side of the gradient
X	X		X	Dipolar vortices, no droplet clustering
	X	X	X	Convective flow, no droplet clustering
X	X	X		Dipolar vortices, no droplet clustering
X		X	X	Convective flow, droplets cluster due to the basic environment
		X	X	Quadrupolar vortices, no droplet clustering

### 2.7 Luminescence Upon Droplet Swarm Clustering

**Figure 7:**
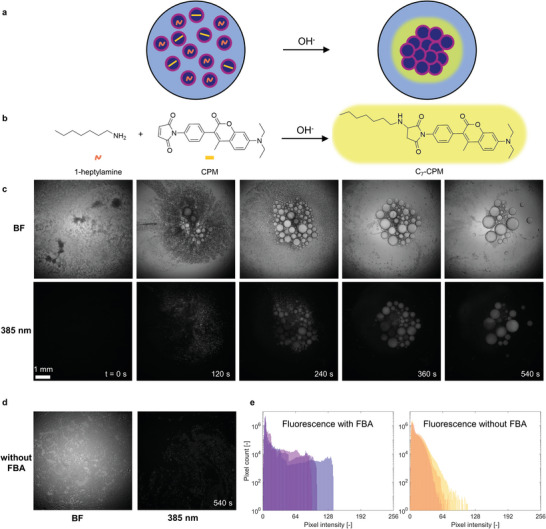
Fluorescence response that is generated upon clustering of the emulsion droplets. a) Two distinct droplet populations are loaded with either 1‐heptylamine or 7‐diethylamino‐3‐(4′‐maleimidylphenyl)−4‐methylcoumarin (CPM). Upon base addition, the droplets merge and subsequently the droplet population becomes fluorescent. b) When the droplet populations merge, 1‐heptylamine and CPM react to form the fluorescent compound C_7_‐FBA. c) Optical microscopy (top row, bright field) and fluorescence microscopy (bottom row, λ_ex_ = 385 nm; λ_em_ >420 nm) on two emulsion droplet populations loaded with 1) 400 mm 1‐heptylamine (3 µL) and 2) 80 mm CPM (1 µL) on an aqueous FBA solution (40 mm) upon application of a base stimulus (2 m NaOH, 7 µL). d) When FBA is omitted from the aqueous solution, the droplets merge less and therefore generate a significantly lower fluorescent intensity response as can be seen from the optical microscopy (left) and fluorescence microscopy (right) images. e) Histograms of fluorescence intensity distribution after 10 min with 40 mm FBA in the aqueous solution (left) and without FBA (right). Shown are 3 replicates for each experiment.

Inspired by *Vibrio fischeri*, which only exhibits bioluminescence when the population density is above a critical density, we prepared a droplet population that exhibits fluorescence when the droplets cluster. Specifically, we employ a strong increase in droplet merging when droplet subpopulations loaded with fluorogenic reagents cluster. In one droplet subpopulation, we include a fluorogenic maleimide, 7‐diethylamino‐3‐(4′‐maleimidylphenyl)−4‐methylcoumarin (CPM). CPM is typically used as a thiol‐reactive dye to quantify thiol concentrations, however, under basic conditions, it can also conjugate with primary amines to form a fluorescent product.^[^ ^48^, ^49^ ^]^ We anticipated that loading 1‐heptylamine to the other droplet subpopulation leads, upon clustering and merging, to the formation of the fluorescent compound C_7_‐CPM (**Figure** **7**a,b).

To assess this concept, we dispersed one subpopulation of emulsion droplets with 1‐heptylamine (400 mm, 3 µL) and another with CPM (80 µm, 1 µL) on top of an aqueous FBA solution (Figure 7c). After ≈60 s, we applied the base stimulus and the droplets responded by clustering. Note that the amount of droplets loaded with 1‐heptylamine is in excess so that sufficient C_7_‐FBA is formed for clustering of all droplets. While clustering, the droplets merge and display fluorescence when irradiated with UV light (385 nm). Over time, the droplets continue to merge until they form a cluster of a few big droplets with a strong fluorescence signal (Figure 7c; Video S8, Supporting Information). Gratifyingly, when FBA was omitted from the aqueous solution, the droplets remained separated (Figure 7d; Video S8, Supporting Information), and even though some droplets merged, the fluorescent intensity obtained after 540 s is much lower, as shown by the fluorescence intensity histograms in Figure 7e.

## 3 Conclusion

We demonstrate a quorum‐sensing mechanism in emulsion droplet swarms. The core of our approach involves the competition between 1) surface tension gradients that are generated locally around the droplets upon release of a surfactant, driving their mutual repulsion, and 2) the release of a surfactant precursor as a signaling molecule from the droplets, that forms, upon a base stimulus, the imine surfactant C_7_‐FBA which cancels the repulsive surface tension gradients. The production of C_7_‐FBA causes a clustering of the droplets and is dependent on the density of the droplets that release the surfactant precursor – enabling quorum‐sensing behavior. Furthermore, the formation of C_7_‐FBA is pH‐dependent, which allows the clustering responses to be manipulated by spatiotemporal base and acid stimuli.

We envision that the concept of quorum sensing in emulsion droplets based on competing surfactant gradients opens a design principle for the field of Systems Chemistry. It allows transforming chemical input signals via dynamic covalent chemistry and surface tension phenomena into droplet swarms with emerging properties at the meso‐ and macro scale.^[^ ^50^, ^51^ ^]^ Chemically controllable competition between attractive and repulsive droplet interactions can be exploited to generate a diverse range of functions that rely on cooperatively operating droplet swarms. For example, in bottom‐up sensor systems based on droplet swarms that explore a substrate for the analyte of interest that is spatially distributed. When a critical number of droplets encounter the analyte, the droplets generate a readout by accumulating at the analyte hotspot. To this end, the droplets need to be loaded with a precursor of the signaling molecule, which gets activated upon reacting with the analyte of interest. Furthermore, the droplet cluster can generate an optical read‐out or combine reagents which in turn activate a chemical probe that enables downstream analysis of the sample. To this end, we note that the clustering of droplets, directed via the population density‐dependent surfactant formation, can be coupled to a diversity of chemical reactions, as we demonstrated with the fluorescent response that was generated upon the merging of two droplet subpopulations. Alternatively, clustering and merging droplets when present at a critical density potentially allows combining reagents that lead to the formation of, e.g., hydrogels or magnetic nanoparticles in ferrofluids (either in the droplets or the aqueous phase): opening new pathways in liquid‐based, nonequilibrium materials.

Ultimately, these concepts can be exploited in autonomously operating droplet swarms that spatiotemporally configure the stimulus that triggers their clustering, expanding on the stimuli that in our system were introduced by external addition of base, or applied as an external pH gradient. For example, clustering droplets can amplify their own production of the stimulus via an autocatalytic reaction, which is triggered if sufficient droplets are in each other's proximity. In this context, we note that *Dictyostelium discoideum* amoebae travel in clusters along weak concentration gradients of cyclic AMP by locally amplifying this gradient through degradation.^[^ ^4^ ^]^ In analogy, synthetic droplets that self‐generate and modulate concentration gradients of stimuli molecules can direct the pathways of swarms along self‐shaped chemoattractant patterns, generating bottom‐up droplet swarms that autonomously navigate their environments.

## 4 Experimental Section

### Materials

1‐heptylamine (99%), Tween 20 (polyoxyethylene (20) sorbitan monolaurate), iodomethane (99%), deuterated sodium hydroxide solution (40 wt.% in D_2_O) and 7‐dimethylamino‐3‐(4′‐maleimidylphenyl)−4‐methylcoumarin (CPM, 95%) were purchased from Sigma Aldrich. Methanol (99.9%), sodium hydroxide (2 m in water), hydrochloric acid (2 m in water), sodium chloride (99.5%), ethanol (99.9%), and 1H,1H,2H,2H‐perfluorooctyltrichlorosilane (97%) were purchased from Fisher Scientific. 1H,1H,2H,2H‐perfluoro‐1‐octanol (98%) was purchased from Fluorochem. 4‐dimethylamino benzaldehyde (98%) and n‐heptane (>99%) were purchased from Merck. Capstone FS‐30 was purchased from SynQuest Labs Inc. Diethyl ether (98%) and 2‐propanol was purchased from VWR. Acetone (99.5%) was purchased from Actu‐All Chemicals. Deuterium oxide (99%) and deuterated hydrochloric acid (20 wt.% in D_2_O) were purchased from Janssen Chimica. Diethyl phthalate (>99%) was purchased from TCI. Sylgard 184 silicone elastomer and Sylgard 184 silicone elastomer curing agent were purchased from Dow. Fluorinert FC‐40 was purchased from Santa Cruz Biotechnology. All materials were used as received.

### Instruments and Settings

For imaging of droplet emulsions on water, an inverted optical microscope (Olympus IX73) was used in bright field mode, equipped with a 1.25x magnification objective (Olympus Plan Apo N 1.25 × 0.04 NA). Grayscale images and movies were acquired at 1 fps with a CMOS camera (Point Grey Grasshopper3). For fluorescence imaging, an inverted fluorescence microscope (Olympus IX83) equipped with a 2x magnification objective (Olympus Plan Apo N 2 × 0.08 NA) was used. Frames were captured in bright field mode and in fluorescence mode, both at one frame per 6 s with an exposure time of 100 and 150 ms, respectively. In fluorescence mode, samples were illuminated with an LED light source (CoolLED pE‐4000) at 385 nm, with an engaged mirror unit (Olympus U‐FUW, excitation filter: 340–390 nm, emission filter: 420 nm – infrared). Grayscale images and movies were acquired with a CMOS camera (Hamamatsu Orca Flash 4.0). Surface tension measurements were performed on a force tensiometer (Biolin Scientific Sigma 701). ^1^H‐NMR measurements were performed on a 400 MHz NMR instrument (Bruker Avance III) and 500 MHz NMR instrument (Bruker Avance III). A pH meter (Mettler Toledo FiveEasy Plus FP20) was used to measure pH. For PDMS and glass bonding, an oxygen plasma cleaner (Diener FEMTO) was used. The mold for the PDMS flow chamber was 3D printed from resin (Liqcreate Clear Impact) using a 3D printer (Creality LD‐002H). After printing, the mold was postprocessed by sequentially washing in 2‐propanol and curing under UV light (385 and 405 nm) in a post‐processing machine (Creality UW‐01). Automated syringe pumps (Cetoni Nemesys B101‐02 E), powered by a base module (Cetoni Base B100‐01 F), were used to inject solutions from glass syringes (1.5 mL, Hamilton) into the PDMS flow chamber.

### Synthesis of 4‐formyl‐N,N,N‐Trimethylbenzenaminium Iodide (FBA)

The synthesis of FBA was carried out according to Yuan et al.^[^ ^52^ ^]^ 4‐dimethylamino benzaldehyde (10 g, 0.0671 mol) and iodomethane (28.57 g, 0.2013 mol) were dissolved in acetone (45 mL) (Figure S1, Supporting Information). The reaction mixture was left overnight at 70 °C in reflux while stirring. The 4‐formyl‐N,N,N‐trimethylbenzenaminium iodide product precipitated, was isolated by filtration, and washed with diethyl ether. A white solid was obtained (7.717 g; 39.6% yield) and characterized with ^1^H‐NMR (400 MHz) and ^13^C‐NMR (400 MHz) in D_2_O (Figure S1, Supporting Information), and with mass spectrometry in methanol. The residual peaks in the ^1^H‐NMR spectrum (singlet at 3.59 ppm, singlet at 6.05 ppm, and quartet at 7.65–7.81 ppm) belong to the hydrated aldehyde (geminal diol), which is present in a 0.2:1 ratio with respect to the normal aldehyde based on the peak integrals. The hydrated aldehyde carbon peaks appear in the ^13^C‐NMR spectrum as well (Figure S1, Supporting Information). MS (ESI) *m/z*: [M + MeOH] ^+^): calcd for C_10_H_14_NO + CH_4_O, 196.3; found, 196.3.

### PDMS Well Preparation

Thin PDMS wells were prepared by mixing silicone elastomer and curing agent in a ratio of 10:1. The solution was degassed for 30 min under vacuum and 2.0 g was transferred to an aluminum dish (100 mm diameter, VWR) and degassed again for 15 min. The solution was cured in a 65 °C oven for 90 min and, after curing, carefully removed from the dish. The resulting PDMS sheet (d = 0.7 mm) was cut in a rectangular shape and four wells were punched with a puncher (12 mm diameter, Gedore). The PDMS wells were carefully cleaned using water and ethanol and any dust was removed using scotch tape (3 m). Next, the PDMS wells were bonded to a glass microscope slide (75 × 50 mm, Corning) by treatment of both the glass surface and the PDMS surface with oxygen plasma for 20 s and subsequently pressing them together.

To make the PDMS wells solvophobic, the following procedure was applied: first, 1.0 mL Fluorinert FC‐40 was placed in a glass vial pre‐heated in a 120 ^○^C oven, degassed by bubbling with argon for 30 s, and 20 µL of 1H,1H,2H,2H‐perfluorooctyltrichlorosilane was added. Then, the PDMS wells were treated with oxygen plasma for 20 s and filled with the silane solution. Finally, the PDMS wells were placed in a 100 °C oven until the silane solution had completely evaporated (typically overnight).

### Quorum Sensing Experiments

Before every quorum sensing experiment, a PDMS well was sequentially washed with MilliQ water and 2‐propanol, dried under pressurized nitrogen, and placed under the microscope. The well (volume = 80 µL) was filled with 120 µL FBA solution to make sure the solution had a convex meniscus. Typically, the solution did not easily spread due to the poor wettability of the solvophobic well and would only spread after the addition of the emulsion droplets due to the decrease in surface tension of the air–water interface. The fluoro‐silane functionalization of the PDMS well (vide supra) was deemed critical to avoid the emulsion droplets getting stuck at the wall of the well. For re‐used PDMS wells where the aqueous solution was observed to spread in the well prior to the addition of the emulsion droplets, it was concluded that the fluoro‐silane functionalization had passed its lifetime and the PDMS well was replaced with a new one.

The emulsion droplets (prepared upon 2 min vortex mixing of the emulsion) were added by a micropipette on top of the aqueous solution in the PDMS well. At a total volume <0.5 µL of emulsion droplets, the droplets are barely visible, while at ≥5 µL the interface of the PDMS well was very crowded and the droplets coalesce rapidly. Hence, total emulsion droplet volumes between 0.5–5 µL were explored.

After the addition of the emulsion droplets by a micropipette, a transparent Petri dish (100 mm diameter) was put over the well to protect the floating emulsion droplets from air convection. The droplets were allowed to spread over the surface of the aqueous solution for ≈30 s, at which point t = 0 is set. Subsequently, the Petri dish cover was removed, the base stimulus was added by a micropipette, and the well was covered again with the Petri dish.

### ^1^H‐NMR Characterization of Imine Amphiphile C_7_‐FBA

FBA (10 mm) and 1‐heptylamine (10 mm) were dissolved in D_2_O. The pH of both solutions was set with DCl and NaOD to either pH 2 or pH 12 and both samples were characterized with ^1^H‐NMR (400 MHz). In accordance with Zhao et al., the aldehyde peak at 10.1 ppm was only visible in the ^1^H‐NMR spectrum of the sample at pH 2, while the imine peak at 8.5 ppm was only visible in the spectrum of the sample at pH 12, see Figure S4 (Supporting Information).^[^ ^40^ ^]^ This means that the imine surfactant C_7_‐FBA only forms under basic conditions.

### Surface Tension Measurements

The surface tension of a solution of Capstone FS‐30 (0.116 wt.%) and FBA (40 mm) at pH 2 was measured in duplicate. Capstone FS‐30 and FBA were dissolved in MilliQ water by vortex mixing and the pH was set to 2 with HCl. A Petri dish (35 mm diameter, Falcon) was filled with 4.5 mL of the sample solution. Measurements were performed with a platinum Wilhelmy plate (wetted length: 39.24 mm) that was cleaned by rinsing with ethanol and heating with a flame torch until glowing red‐hot. The Wilhelmy plate was prewetted in the solution and subsequently immersed to a depth of 3.5 mm. The surface tension was monitored continuously for 3 min at a sample rate of 1 Hz.

The surface tension of the following aqueous solutions was measured in triplicate at pH 2 and pH 12: 1) FBA (40 mm) and 1‐heptylamine (10 mm); 2) FBA (40 mm), 1‐heptylamine (10 mm) and Tween 20 (0.375 mm); and 3) Tween 20 (0.375 mm). Furthermore, the surface tension of FBA (40 mm) and 1‐heptylamine (2.5, 5, 7.5 mm) was measured in triplicate at pH 12. This concentration of Tween 20 was chosen as representative for the quorum sensing experiments because it was the upper limit to the concentration of Tween 20 in any experiment performed for this paper: assume 5 µL emulsion (10/90 v/v% aqueous phase/organic phase) fully mixes with the 120 µL solution in the PDMS well, then the concentration Tween 20 was 0.375 mm. For the surface tension measurements, a Petri dish (35 mm, Falcon) was filled with 7.0 mL sample. Measurements were performed with a platinum Du Noüy ring (120.4 mm wetting length) that was cleaned by rinsing with ethanol and heating with a flame torch until glowing red‐hot. Twenty data points were collected over the course of ≈5 min and the average value was taken as the surface tension.

### pH Dependent 1‐heptylamine Transfer from Emulsion Droplets to Aqueous Solution

A mixture of 167 µL organic phase (80/20 v/v% 1‐octanol/perfluorinated octanol) loaded with 1‐heptylamine (400 mm) and 1500 µL D_2_O was prepared by vortex mixing. The pH of the aqueous phase was measured to be 11.0. Subsequently, three samples were prepared: sample 1 was kept at pH 11.0, sample 2 was adjusted to pH 3.0 with deuterated hydrochloric acid (20 wt.% DCl in D_2_O), and sample 3 was adjusted to pH 12.5 with deuterated sodium hydroxide (40 wt.% NaOD in D_2_O). The mixtures were vortexed thoroughly for 1 min and left to equilibrate for 30 min. Then, the samples were centrifuged to separate the phases, and 1.0 mL of the aqueous phase was carefully transferred to an NMR tube. Methanol (10 mm) was added as an internal standard and the samples were characterized with ^1^H‐NMR (500 MHz). To quantify the concentration of 1‐heptylamine that has partitioned into the aqueous phase, the integration of the ─CH_3_ peak of methanol (3.28 ppm) was compared to the integration of the peaks of the protons that belong to the α‐position of 1‐heptylamine (i.e., NH_2_‐C ***H***_2_‐C_6_H_13_, Figure S4, Table S1, Supporting Information). It was noted that the upfield shift of these protons with increasing pH is due to the decrease in protonation of the amine group. The NH_2_‐C ***H***_2_‐C_6_H_13_ proton peak positions and integral values are shown in Table S1 (Supporting Information), together with the calculated concentration of 1‐heptylamine based on the integral value.

### Image Analysis Algorithm for Emulsion Droplet Clustering

To quantify the extent of droplet clustering, a Matlab script was written in which the distribution of droplets around the “center of mass” was computed. This section provides a detailed description of all computational steps involved. The used Matlab functions were italicized. A visual overview of all computational steps is provided in Figure S7, (Supporting Information).

Step 1: The first frames of each raw video were discarded, up to the point where the base stimulus had been added to the well and the lid was placed back on top of the PDMS well to prevent airflow from perturbing the droplets.
Step 2: To exclude all pixels that are not part of the sample well, a binary mask was calculated based on the large contrast between the bright sample well and the dark PDMS layer outside the well. The first frame was binarized (*imbinarize*) using Otsu's threshold^[^ ^53^ ^]^ resulting in well pixels flagged with a 1 and pixels outside the well flagged by a 0. Subsequently, all regions of connected pixels with value 1 were computed (*regionprops*), and the largest region was selected and visually confirmed to represent the sample well. Due to the presence of droplets in the sample well, all holes in the binary mask were filled (*imfill*) to end up with a binary mask with the same shape as the sample well. Then, the binary mask was overlaid on each nondiscarded frame (see step 1) to mask the pixels outside the sample well in these frames.
Step 3: To calculate the center of mass of the emulsion droplets in each frame, the color scale for each frame was inverted, which resulted in pixels belonging to emulsion droplets gaining high‐intensity values (i.e., appearing as white structures on a dark background). Subsequently, to increase the contrast between the emulsion droplets and the background, each frame was binarized (*imbinarize*) using Otsu's threshold. Finally, the center of mass (*x*_c.o.m._,*y*_c.o.m._) of the emulsion droplets was calculated using the following equation
(1) $$x_{c.o.m.}=\frac{\sum x_{i}\cdot I_{i}}{\sum I_{i}}\;,\;y_{c.o.m.}=\frac{\sum y_{i}\cdot I_{i}}{\sum I_{i}}$$

in which *x*_i_,*y*_i_ and *I*_i_ are the x coordinate, y coordinate, and intensity value of pixel *i*, respectively. Note that only the nonmasked pixels are considered in the summations.

Step 4: The cumulative pixel intensity Σ*I* was calculated as a function of the Euclidian distance from the center of mass, $r\;={\left(\Delta x_{i}^{2}+\Delta y_{i}^{2}\right)}^{1/2\;}\;$, where Δ *x*_i_ = *x*_c.o.m._  − *x*_i_ and Δ *y*_i_ = *y*_c.o.m._  −*y*_i_. To this end, all pixel intensity values were sorted in an array based on their Euclidian distance *r* to the center of mass (from low to high *r*) and the cumulative pixel intensity was computed (*cumsum*). The cumulative intensity values were normalized by the total sum of pixel intensity so that these values range from 0 to 1. Furthermore, to compensate for differences in the amount of well pixels between experiments, the distance *r* was normalized by the maximum distance *r_max_*.
Step 5: To differentiate between clustered and nonclustered droplet swarms, the previously calculated binary well mask was taken as an entirely homogeneous reference frame. The cumulative pixel intensity was calculated for this reference frame, normalized, and subsequently subtracted from the cumulative pixel intensity for each video frame, to give a difference in cumulative pixel intensity Δ$\bar{\Sigma I}$($\bar{r}$). Finally, a droplet density ρ_*I*_ was calculated through the division of the difference cumulative pixel intensity by the normalized pixel intensity sum of the reference frame (examples in Figure S5b,c, Supporting Information). Then, the clustering parameter for each frame was calculated by integrating this value as a function of the normalized distance $\bar{r}=\;r/r_{\max}\;$(*trapz*). For all experiments, the value of the resulting clustering parameter varied between −0.3, indicating that the emulsion droplets are spread out, and 0.3, indicating that the emulsion droplets are clustered together. As a final measure of the clustering parameter (*CP*), the maximum integral value for the entire video was considered.

### PDMS Flow Chamber Preparation

To introduce a spatial pH gradient in the well with the emulsion droplets, a PDMS device was designed that allows for the injection of acid and base on opposite sides of a circular well which has the same diameter (12 mm) as the previously used sample well.

First, a device mold was designed to cure the PDMS, of which the exact dimensions are provided in Figure S8 (Supporting Information). This mold was 3D printed and cleaned with n‐heptane to remove all residues of any resin crosslinker left after printing. A mixture of silicone elastomer and curing agent (10:1) was prepared, thoroughly mixed, and degassed under vacuum for 30 min, and 2.0 g of this mixture was added to the device mold. After curing for 90 min in a 65 °C oven, the PDMS device (1.5 mm thick) was carefully removed using a spatula. To create the sample well, a hole was punched with a puncher (12 mm diameter, Gedore) and three in/outlets were punched (3 mm diameter, Biopunch). The PDMS device was carefully cleaned using water and ethanol and any dust was removed using scotch tape (3 m). Then the PDMS device was bonded to a glass microscope slide (75 × 50 mm, Corning) by treatment of both the glass surface and the PDMS surface with oxygen plasma for 20 s and subsequently pressing them together.

To create support for tubing, three PDMS cylinders were bonded (using oxygen plasma treatment as described above) on top of the in‐ and outlets, see Figure S8 (Supporting Information). These PDMS cylinders were prepared from a slab of PDMS (8 mm thick) made by curing a 23.0 g PDMS mixture in an aluminum dish (100 mm diameter, VWR) cured for 2.5 h in a 65 °C oven. A channel was punched in these support cylinders (1 mm diameter, Biopunch) to guide the tubing to the in/outlets. The PDMS device was subsequently made solvophobic, following the same procedure as described for the PDMS well (vide supra).

### Spatial pH Gradient Experiments

Before every experiment, the PDMS flow chamber was thoroughly rinsed with water and 2‐propanol and dried under pressurized nitrogen. PTFE tubing (outer diameter 1 mm) was cut to size, carefully inserted into the PDMS flow chamber, and connected to glass syringes filled with base (2 m NaOH) or acid (2 m HCl), while the outlet syringe was left empty. The flow was tested by pumping (flow rate = 50 µL s^−1^) 300 µL base into the chamber to make sure that the whole chamber (volume chamber = 170 µL) was filled. Then, the base was removed from the outlet syringe (flow rate = 50 µL s^−1^). The same procedure was followed for the acid. Finally, the well was rinsed three times by adding 300 µL water with a micropipette and subsequentially removing the water from the well with the micropipette until no liquid was left in the chamber.

For each experiment, 160 µL FBA solution (40 mm, pH 2) was added to the well with a micropipette. Similar to the PDMS well, the lifetime of the PDMS flow chamber was inspected based on the wettability of the chamber by the aqueous solution. If the chamber was fully wetted by the aqueous solution, a fresh PDMS chamber was prepared.

Subsequently, the emulsion droplets (2 µL) were added with a micropipette on top of the aqueous solution, and a Petri dish lid (100 mm diameter) with three drilled holes for the PTFE tubing was put on top of the PDMS chamber to cover it from air convection. A lower emulsion volume (2 µL instead of 4 µL) was chosen to avoid the clustering of droplets at the periphery of the rotating vortices sticking to the edge of the well. The droplets were left to spread for ≈30 s after which the flow profile was started: +90 µL h^−1^ flow of acid and base and −180 µL h^−1^ flow to the outlet syringe, so that the total retention time was ≈50 min. After 15 min, the dilution of the droplets was clearly visible and the flow was halted.

### Particle Image Velocimetry (PIV) Analysis

To visualize the convection and clustering dynamics in the experiments in which a spatial pH gradient is applied to the emulsion droplets, PIV analysis was performed using the Matlab plugin PIVlab.^[^ ^46^ ^]^ Although PIV is typically used to measure the movement of tracer particles with large optical contrast to the fluid in which they are dispersed, the contrast between the emulsion droplets and the aqueous solution is large enough to get consistent results. For each experiment, 120 consecutive frames were analyzed and the mean velocity and direction of the emulsion droplets in this time frame (120 s) were calculated. In PIVlab the following steps were performed:

First, the area outside the flow chamber, including the three connecting channels, was masked by tracing the well edges. This mask was applied to all frames that were analyzed. The contrast for each frame was optimized using the inbuilt CLAHE algorithm (Contrast Limited Adaptive Histogram Equalization) with the neighborhood region size set to 64 pixels.

For the analysis, the FFT window deformation mode was selected and three passes were performed. For pass one, the interrogation area was set to 64 pixels with a step size of 32 pixels, and both the area and step size were subsequently halved in the next two passes. The sub‐pixel estimator was set to Gaussian 2 × 3 points and the correlation robustness to standard. The pixel length was measured to be 0.00705 mm and the time step was set to the camera frame rate of 1 s. For postprocessing, vector validation was performed with a standard deviation filter set to a threshold of 8, and a local median filter set to a threshold of 3. Finally, the velocity magnitude, averaged over all 120 frames, was calculated for every 80 × 80 pixel region.

## Conflict of Interest

The authors declare no conflict of interest.

## Author Contributions

P.d.V. performed methodology, investigation, formal analysis, writing original draft, review, and editing. D.K. performed methodology, investigation, review, and editing. A.‐D.N. performed conceptualization, review, and editing. P.K. performed supervision, conceptualization, writing original draft, review, and editing.

## Supporting information

Supporting Information

Supplemental Video 1

Supplemental Video 2

Supplemental Video 3

Supplemental Video 4

Supplemental Video 5

Supplemental Video 6

Supplemental Video 7

Supplemental Video 8

## Data Availability

The data that support the findings of this study are available in the supplementary material of this article.

## References

[1] B. L. Bassler , R. Losick , Cell 2006, 125, 237. 16630813 10.1016/j.cell.2006.04.001

[2] C. M. Waters , B. L. Bassler , Annu. Rev. Cell Dev. Biol. 2005, 21, 319. 16212498 10.1146/annurev.cellbio.21.012704.131001

[3] T. Gregor , K. Fujimoto , N. Masaki , S. Sawai , Science 2010, 328, 1021. 20413456 10.1126/science.1183415 PMC3120019

[4] L. Tweedy , P. A. Thomason , P. I. Paschke , K. Martin , L. M. Machesky , M. Zagnoni , R. H. Insall , Science 2020, 369, aay9792. 10.1126/science.aay9792 32855311

[5] K. L. Visick , E. V. Stabb , E. G. Ruby , Nat. Rev. Microbiol. 2021, 19, 654. 34089008 10.1038/s41579-021-00557-0 PMC8529645

[6] T. Kojima , H. Kitahata , K. Asakura , T. Banno , Cell Rep. Phys. Sci. 2023, 4, 101222.

[7] C. M. Wentworth , A. C. Castonguay , P. G. Moerman , C. H. Meredith , R. V. Balaj , S. I. Cheon , L. D. Zarzar , Angew. Chem., Int. Ed. 2022, 61, 202204510. 10.1002/anie.202204510 35678216

[8] J. Vialetto , M. Anyfantakis , S. Rudiuk , M. Morel , D. Baigl , Angew. Chem. Int. Ed Engl. 2019, 58, 9145. 31041837 10.1002/anie.201904093

[9] T. Bäuerle , A. Fischer , T. Speck , C. Bechinger , Nat. Commun. 2018, 9, 3232. 30104679 10.1038/s41467-018-05675-7 PMC6089911

[10] M. Ibele , T. E. Mallouk , A. Sen , Angew. Chem. Int. Ed Engl. 2009, 48, 3308. 19338004 10.1002/anie.200804704

[11] H. Niederholtmeyer , C. Chaggan , N. K. Devaraj , Nat. Commun. 2018, 9, 5027. 30487584 10.1038/s41467-018-07473-7 PMC6261949

[12] S. Basu , Y. Gerchman , C. H. Collins , F. H. Arnold , R. Weiss , Nature 2005, 434, 1130. 15858574 10.1038/nature03461

[13] A. F. Taylor , M. R. Tinsley , F. Wang , Z. Huang , K. Showalter , Science 2009, 323, 614. 19179525 10.1126/science.1166253

[14] T. Bánsági Jr , A. F. Taylor , J. R. Soc. Interface 2017, 15, 945. 10.1098/rsif.2017.0945 PMC5905404 29514986

[15] V. M. Markovic , T. Bánsági Jr , D. McKenzie , A. Mai , J. A. Pojman , A. F. Taylor , Chaos 2019, 29, 033130. 30927847 10.1063/1.5089295

[16] R. Seemann , J.‐B. Fleury , C. C. Maass , Eur. Phys. J. Spec. Top. 2016, 225, 2227.

[17] Z. Izri , M. N. van der Linden , S. Michelin , O. Dauchot , Phys. Rev. Lett. 2014, 113, 248302. 25541808 10.1103/PhysRevLett.113.248302

[18] T. Banno , Y. Tanaka , K. Asakura , T. Toyota , Langmuir 2016, 32, 9591. 27580350 10.1021/acs.langmuir.6b02449

[19] P. Kumar , D. Horváth , Á. Tóth , Soft Matter 2023, 19, 4137. 37249219 10.1039/d3sm00461a

[20] A.‐D. C. Nguindjel , P. A. Korevaar , ChemSystemsChem 2021, 3, 2100021.

[21] G. Lu , G. Zhu , Q. Zhang , P. Tian , M. Cheng , F. Shi , Angew. Chem. Int. Ed. Engl. 2023, 62, e202300448. 36786533 10.1002/anie.202300448

[22] S. Thutupalli , R. Seemann , S. Herminghaus , New J. Phys. 2011, 13, 073021.

[23] D. Vella , L. Mahadevan , Am. J. Phys. 2005, 73, 817.

[24] L. W. Honaker , C. Chen , F. M. H. Dautzenberg , S. Brugman , S. Deshpande , ACS Appl. Mater. Interfaces 2022, 14, 37316. 35969154 10.1021/acsami.2c06923 PMC9412956

[25] B. J. Ortiz , M. E. Boursier , K. L. Barrett , D. E. Manson , D. Amador‐Noguez , N. L. Abbott , H. E. Blackwell , D. M. Lynn , ACS Appl. Mater. Interfaces 2020, 12, 29056. 32484648 10.1021/acsami.0c05792 PMC7343617

[26] X. Liu , N. Kent , A. Ceballos , R. Streubel , Y. Jiang , Y. Chai , P. Y. Kim , J. Forth , F. Hellman , S. Shi , D. Wang , B. A. Helms , P. D. Ashby , P. Fischer , T. P. Russell , Science 2019, 365, 264. 31320536 10.1126/science.aaw8719

[27] N. Denkov , S. Tcholakova , I. Lesov , D. Cholakova , S. K. Smoukov , Nature 2015, 528, 392. 26649824 10.1038/nature16189

[28] A. E. Goodling , S. Nagelberg , B. Kaehr , C. H. Meredith , S. I. Cheon , A. P. Saunders , M. Kolle , L. D. Zarzar , Nature 2019, 566, 523. 30814712 10.1038/s41586-019-0946-4

[29] I. Lagzi , S. Soh , P. J. Wesson , K. P. Browne , B. A. Grzybowski , J. Am. Chem. Soc. 2010, 132, 1198. 20063877 10.1021/ja9076793

[30] D. Babu , R. J. H. Scanes , R. Plamont , A. Ryabchun , F. Lancia , T. Kudernac , S. P. Fletcher , N. Katsonis , Nat. Commun. 2021, 12, 2959. 34011926 10.1038/s41467-021-23022-1 PMC8134444

[31] C. H. Meredith , P. G. Moerman , J. Groenewold , Y. J. Chiu , W. K. Kegel , A. van Blaaderen , L. D. Zarzar , Nat. Chem. 2020, 12, 1136. 33199888 10.1038/s41557-020-00575-0

[32] M. Tena‐Solsona , C. Wanzke , B. Riess , A. R. Bausch , J. Boekhoven , Nat. Commun. 2018, 9, 2044. 29795292 10.1038/s41467-018-04488-y PMC5966463

[33] M. Matsuo , H. Hashishita , S. Tanaka , S. Nakata , Langmuir 2023, 39, 2073. 36692295 10.1021/acs.langmuir.2c03344

[34] M. Winkens , P. A. Korevaar , Langmuir 2022, 38, 10799. 36005886 10.1021/acs.langmuir.2c01241 PMC9454263

[35] M. Winkens , A. Vilcan , P. J. de Visser , F. V. de Graaf , P. A. Korevaar , Small 2023, 19, e2206800. 36799188 10.1002/smll.202206800

[36] B. Kichatov , A. Korshunov , V. Sudakov , V. Gubernov , A. Golubkov , A. Kiverin , Langmuir 2021, 37, 9892. 34347492 10.1021/acs.langmuir.1c01615

[37] J. Čejková , K. Schwarzenberger , K. Eckert , S. Tanaka , Colloids Surf. A Physicochem. Eng. Asp. 2019, 566, 141.

[38] L. Wang , Y. Lin , Y. Zhou , H. Xie , J. Song , M. Li , Y. Huang , X. Huang , S. Mann , Angew. Chem. Int. Ed Engl. 2019, 131, 1079. 10.1002/anie.201812111 30480856

[39] Z. Yang , J. Wei , Y. I. Sobolev , B. A. Grzybowski , Nature 2018, 553, 313. 29320473 10.1038/nature25137

[40] W. Zhao , H. Wang , Y. Wang , Soft Matter 2018, 14, 4178. 29740650 10.1039/c8sm00773j

[41] C. A. Zentner , F. Anson , S. Thayumanavan , T. M. Swager , J. Am. Chem. Soc. 2019, 141, 18048. 31674769 10.1021/jacs.9b06852

[42] G. Ren , L. Wang , Q. Chen , Z. Xu , J. Xu , D. Sun , Langmuir 2017, 33, 3040. 28282144 10.1021/acs.langmuir.6b04546

[43] S. Soh , K. J. M. Bishop , B. A. Grzybowski , J. Phys. Chem. B 2008, 112, 10848. 18686988 10.1021/jp7111457

[44] T. Banno , A. Asami , N. Ueno , H. Kitahata , Y. Koyano , K. Asakura , T. Toyota , Sci. Rep. 2016, 6, 31292. 27503336 10.1038/srep31292 PMC4977503

[45] N. D. Vassileva , D. van den Ende , F. Mugele , J. Mellema , Langmuir 2005, 21, 11190. 16285790 10.1021/la051186o

[46] W. Thielicke , R. Sonntag , J. Open Res. Softw. 2021, 9, 12.

[47] S. Maiti , O. E. Shklyaev , A. C. Balazs , A. Sen , Langmuir 2019, 35, 3724. 30721619 10.1021/acs.langmuir.8b03607

[48] T. O. Sippel , J. Histochem. Cytochem. 1981, 29, 314. 7019305 10.1177/29.2.7019305

[49] F. C. Ayers , G. L. Warner , K. L. Smith , D. A. Lawrence , Anal. Biochem. 1986, 154, 186. 3706721 10.1016/0003-2697(86)90513-0

[50] A.‐D. C. Nguindjel , P. J. de Visser , M. Winkens , P. A. Korevaar , Phys. Chem. Chem. Phys. 2022, 24, 23980. 36172850 10.1039/d2cp02542f PMC9554936

[51] A. Walther , Adv. Mater. 2020, 32, 1905111

[52] H. Yuan , Z. Zhou , J. Xiao , L. Liang , L. Dai , Tetrahedron Asymmetry 2010, 21, 1874.

[53] N. Otsu , IEEE Trans. Syst. Man Cybern. 1979, 9, 62. 10.1109/tsmc.1979.4310068 10242122

