# Supplementary material for: Quorum Sensing in Emulsion Droplet Swarms Driven by a Surfactant Competition System
Source: Adv Sci (Weinh). 2024 Jun 17;11(30):2307919. doi: 10.1002/advs.202307919 (PMC11321703; doi:10.1002/advs.202307919)
Supplement: Supplementary file 1 — Supporting Information [file ADVS-11-2307919-s006.docx]

Supporting Information

Quorum Sensing in Emulsion Droplet Swarms Driven by a Surfactant Competition System.

*Pieter J. de Visser, Dimitrios Karagrigoriou, Anne-Déborah C. Nguindjel & Peter A. Korevaar**

**Table of Contents**

Supporting Section 1: Estimation Magnitude Surface Tension Gradient

Figure S1: FBA Synthesis

Figure S2: Control Experiments Quorum Sensing Experiments

Figure S3: Control Experiments Quorum Sensing Experiments

Figure S4: NMR C_7_-FBA

Figure S5: pH Dependent Partitioning 1-heptylamine

Table S1: pH Dependent Partitioning 1-heptylamine

Figure S6: Control Experiments Spatial pH Gradient

Figure S7: Image Analysis Algorithm

Figure S8: PDMS Flow Chamber Preparation

Description of Videos S1 – S8

**Supporting Section 1.** **Estimation Magnitude Surface Tension Gradient**

We attempted to estimate the surface tension gradient involved, via the velocity *v* of the cluster front. Right after the addition of base, the front moves with a velocity *v* = 2 mm s^-1^. Via the relationship $\eta\frac{\partial v}{\partial z}=\frac{\partial\gamma}{\partial x}$, we can approximate the surface tension gradient $\frac{\partial\gamma}{\partial x}$, with viscosity *η* = 1 mPa.s for an aqueous solution and estimating a velocity *v* = 0 at z = –0.35 mm from the air-water interface. With *v* = 2 mm s^-1^, we obtain $\frac{\partial\gamma}{\partial x}$ = 5.7 mN m^-2^, which would imply a surface tension difference Δ*γ* = 0.06 mN m^-1^ over the length of the well (approx. 1 cm) between the C_7_-FBA-rich and C_7_-FBA-poor region.

To overcome the capillary force amongst the clustered droplets, we approximate the Marangoni force *F*_Mar_ to be proportional to the difference in surface tension force exerted at one side of the droplet (*F*_1_ = *γ*_1_∙*d*) and the other side (*F*_2_ = *γ*_2_∙*d*). Hence, *F*_Mar_ = *F*_1_ – *F*_2_ = (*γ*_1_ – *γ*_2_)∙*d* = Δ*γ*∙*d*, with Δ*γ* the surface tension gradient and *d* the diameter of the droplet.


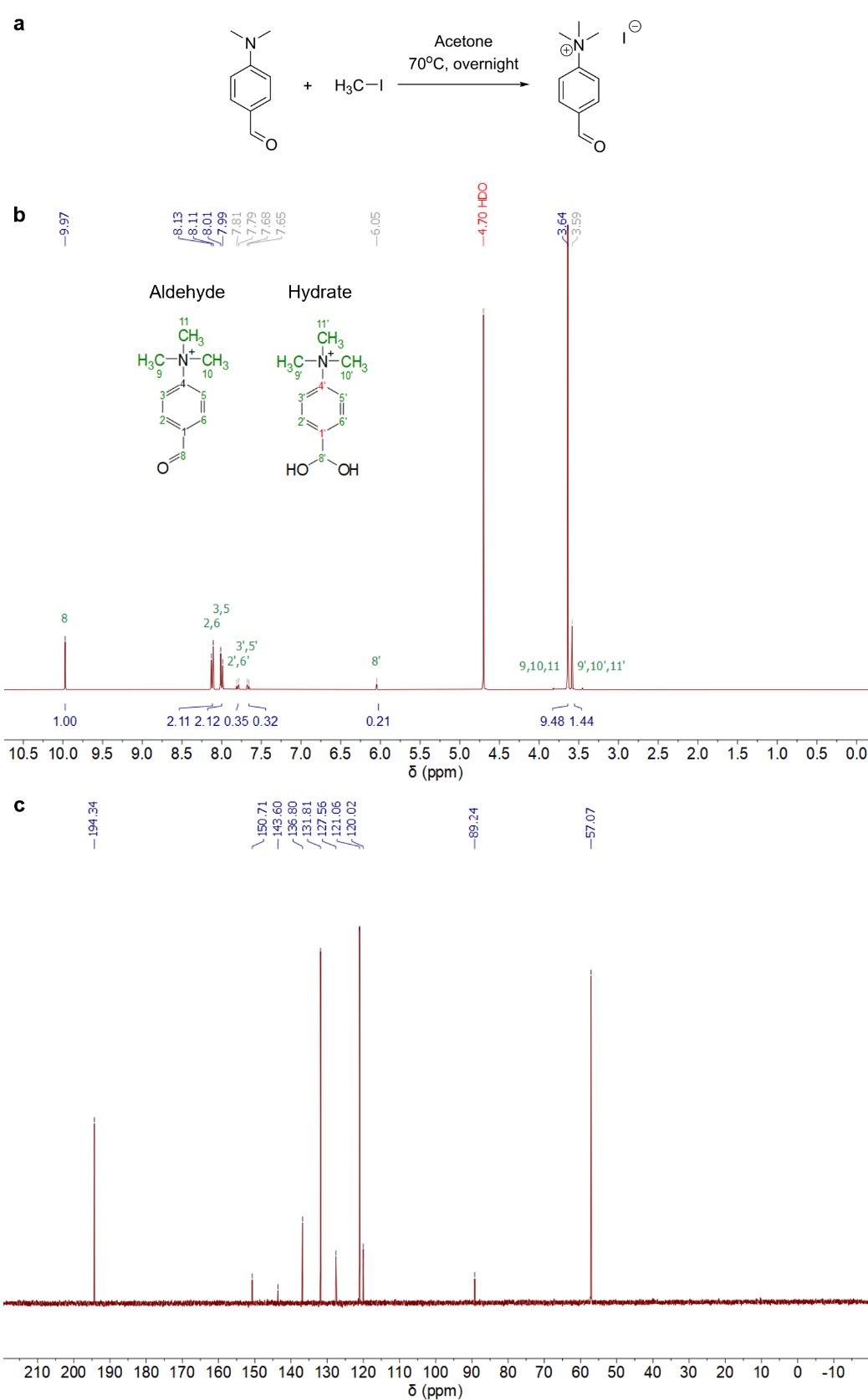


**Figure S1.** a) Reaction scheme of the synthesis of 4-formyl-N,N,N-trimethylbenzenaminium iodide (FBA). b) ^1^H-NMR spectrum of FBA in D_2_O. The hydrated aldehyde (geminal diol) protons are marked with an apostrophe (‘). c) ^13^C-NMR spectrum of FBA in D_2_O.


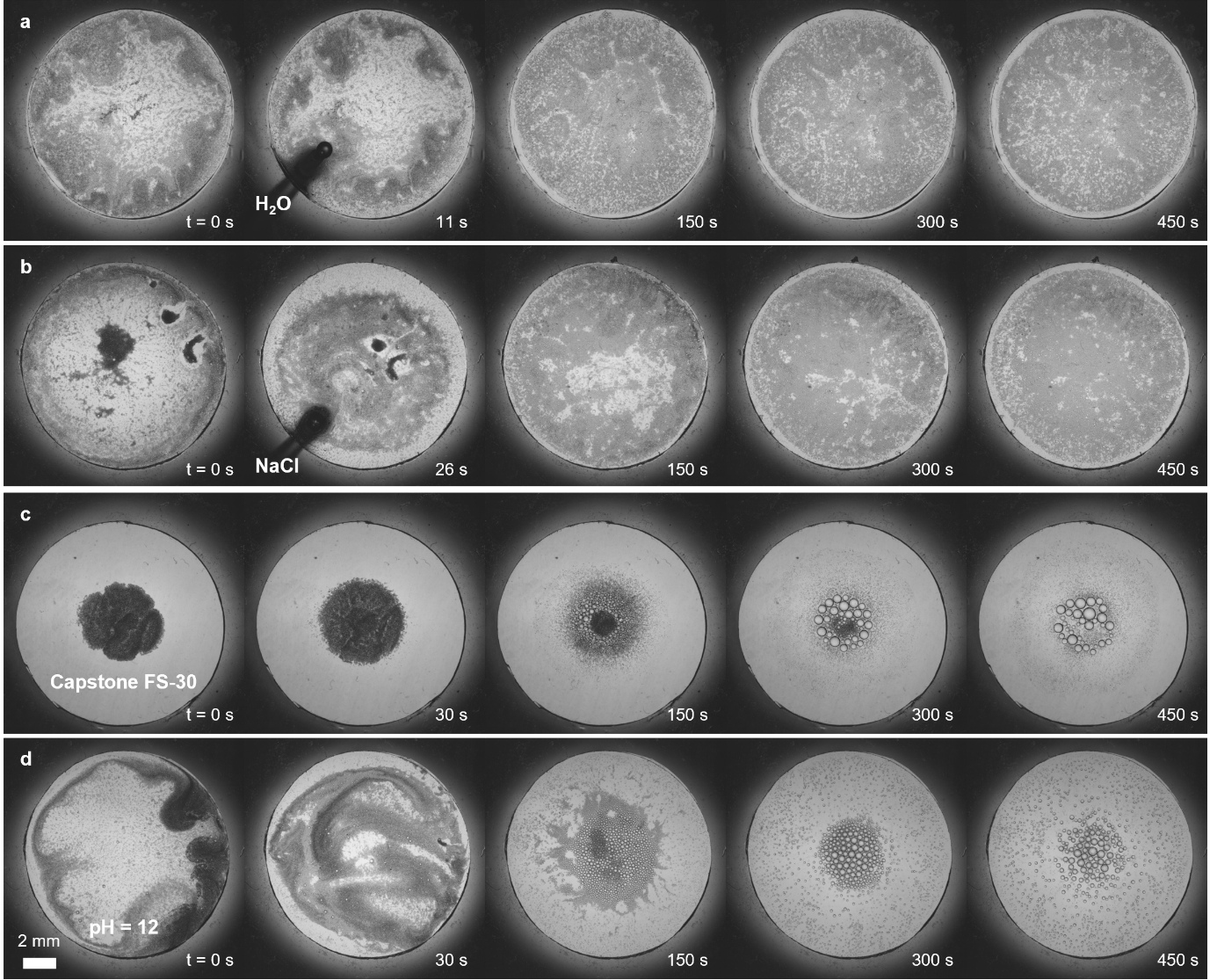


**Figure S2.** Optical microscopy images of emulsion droplets (4 μL, 400 mm 1-heptylamine) in 4 different control experiments. a) Water (MilliQ, 7 μL) is added instead of base. Upon addition of water, the droplets do not cluster. b) 2 m NaCl (7 μL) is added instead of base. Upon addition of the salt solution, the droplets do not cluster. c) Capstone FS-30 (0.116 wt%) is dissolved in the aqueous solution (pH 2). The emulsion droplets cluster immediately when applied at the air-water interface and do not spread. d) The initial pH of the aqueous solution is set to 12.0 and no base is added. The emulsion droplets cluster slightly and spread as time progresses.


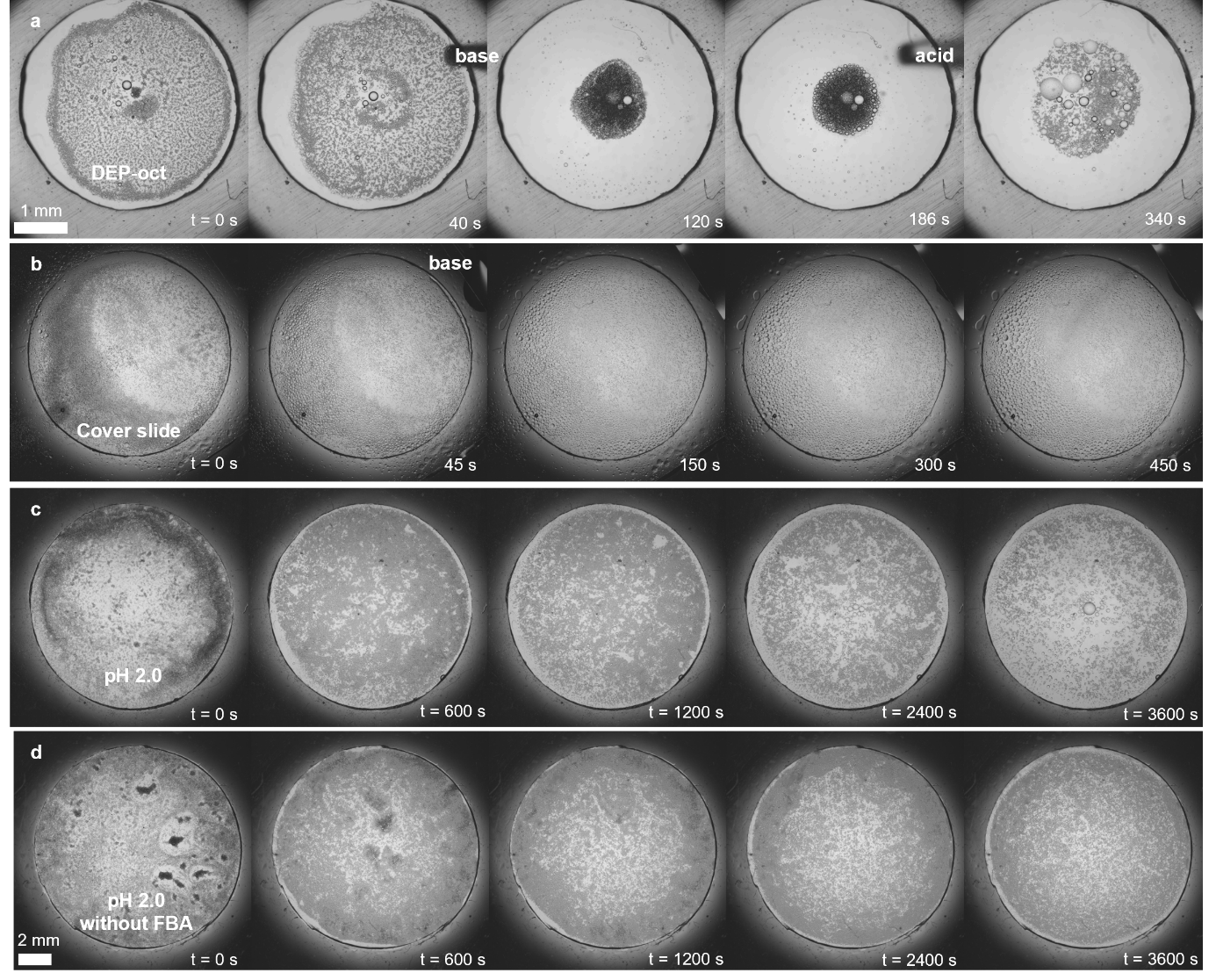
**Figure S3.** Optical microscopy images of emulsion droplets in 4 different control experiments showing the effect of varying the droplet composition (a), covering the air-water interface (b), and the long-term stability of non-clustered droplet swarms with and without FBA in the aqueous solution (c-d). a) Emulsion droplets (0.5 μL, 400 mm 1-heptylamine) composed of 1-octanol and diethyl phthalate (DEP) (45:55 v/v%) were deposited on an aqueous FBA solution (65 μL, 40 mm) in a smaller PDMS well (6 mm diameter). Upon subsequent base and acid addition, the droplets cluster and spread like the emulsion droplets composed of 1-octanol and perfluorinated octanol. b) After deposition of the emulsion droplets (4 μL, 400 mm 1-heptylamine) on the aqueous solution, the PDMS well was covered with a glass cover slide (t = 0 s) to remove the air-water interface. The droplets stick to the glass slide and do not respond to the addition of base from the side at t = 45 s. c-d) After 1h at pH 2, more droplets have merged in the droplet swarm (4 μL, 400 mm 1-heptylamine) when FBA (40 mm) is present in the aqueous solution (c) compared to when FBA is absent from the aqueous solution (d).


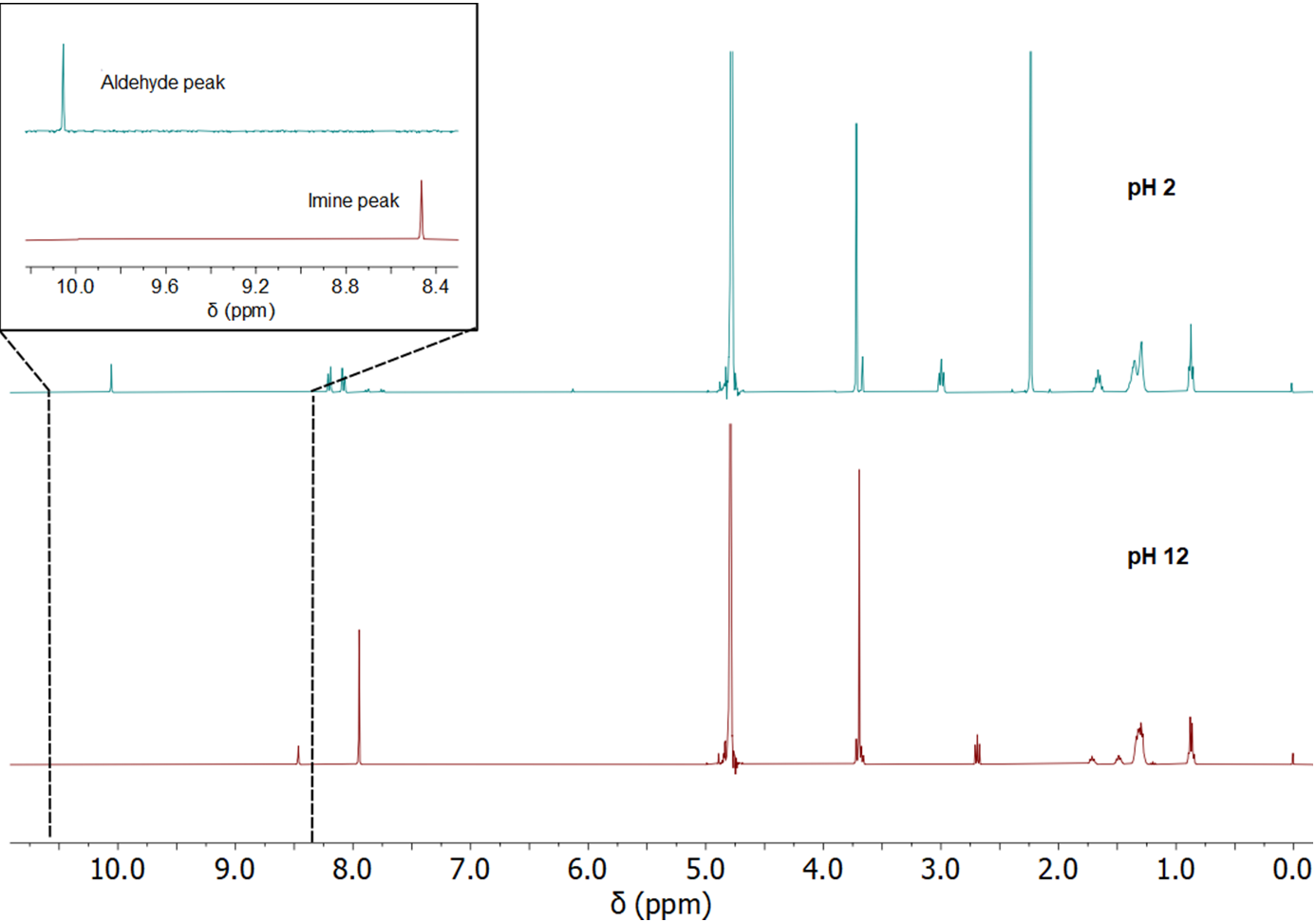


**Figure S4.** ^1^H-NMR spectra of imine amphiphile at pH 2 and pH 12. Inset shows zoom-in to the region with the imine and aldehyde peaks.

**
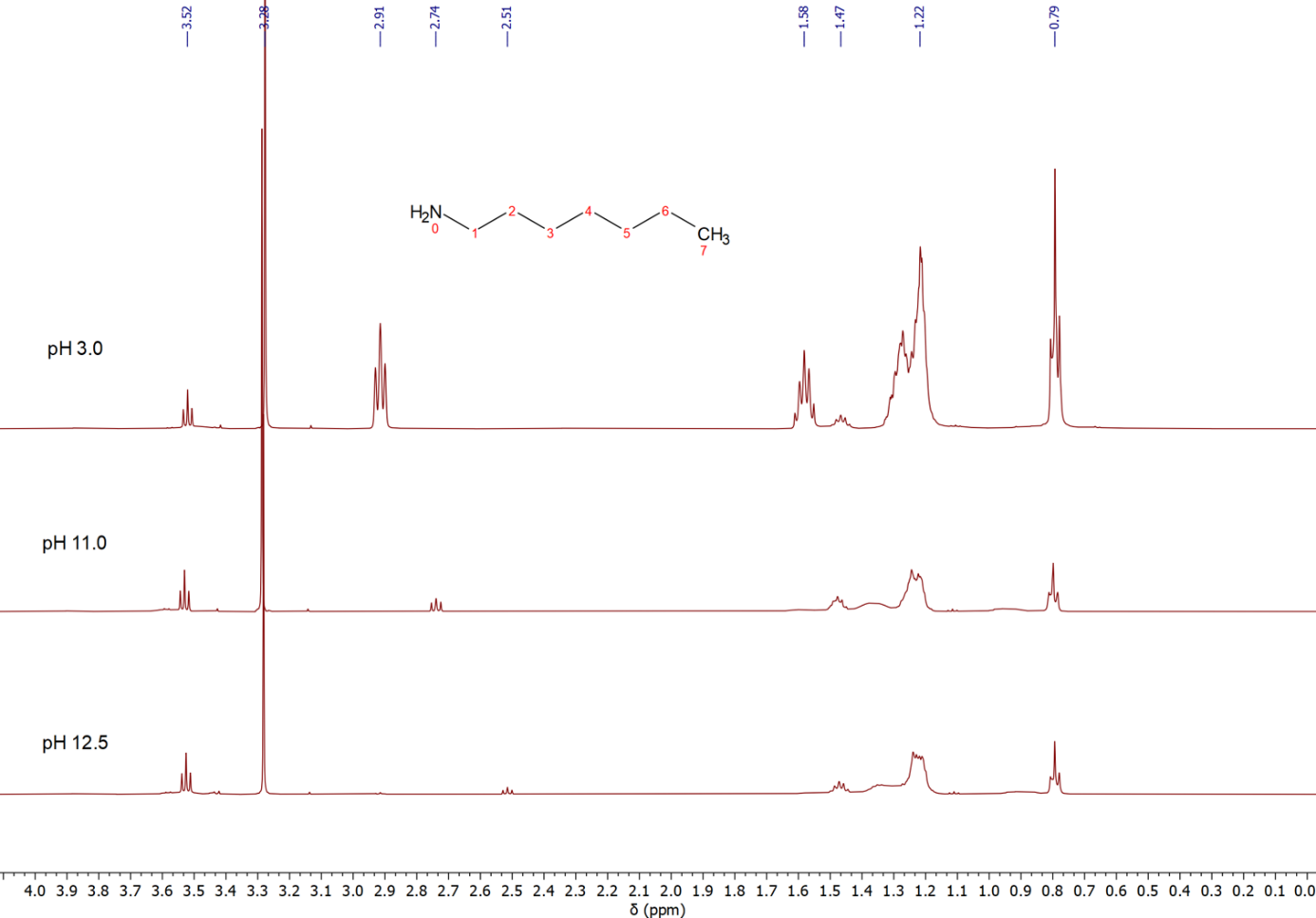
**

**Figure S5.** ^1^H-NMR spectra of 1-heptylamine after partitioning from organic phase (167 μL, 400 mm 1-heptylamine) into aqueous phase (1500 μL) at pH 3.0, pH 11.0, and pH 12.5.

**Table S1.** NH_2_-C*H*_2_-C_6_H_13_ proton peak positions in NMR spectrum, NMR peak integral values and concentrations calculated from these integral peak values at pH 3.0, 11.0 and 12.5.

| pH | Shift  [ppm] | Integral  [-] | Concentration 1-heptylamine  [mm] |
| --- | --- | --- | --- |
| 3.0 | 2.91 | 2.79 | 14 |
| 11.0 | 2.74 | 0.29 | 1.45 |
| 12.5 | 2.51 | 0.15 | 0.75 |


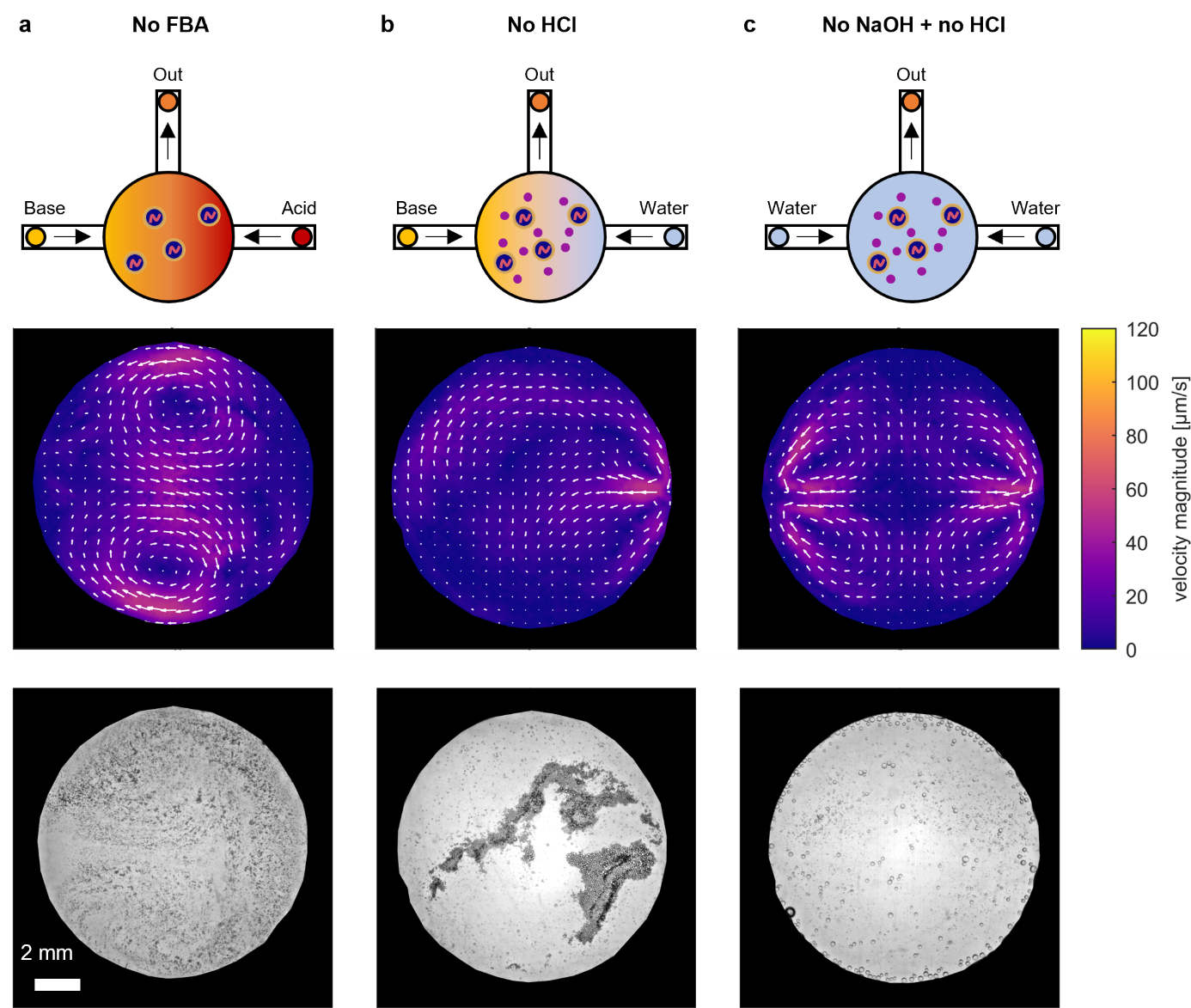


**Figure S6.** Spatiotemporal droplet population dynamics in control experiments of droplets loaded with 1-heptylamine (400 mm). The top row outlines the configuration of the acid and base inlets; the middle row shows the flow profiles in the well (acquired by PIV analysis) and the bottom row shows optical microscopy images of the droplet populations. a) The aqueous solution does not contain FBA; base (2 m NaOH) and acid (2 m HCl) are injected into the chamber. b) The aqueous solution contains FBA (40 mm, pH 2), while base is injected into the chamber. c) The aqueous solution contains FBA, and neither acid nor base are injected into the chamber.


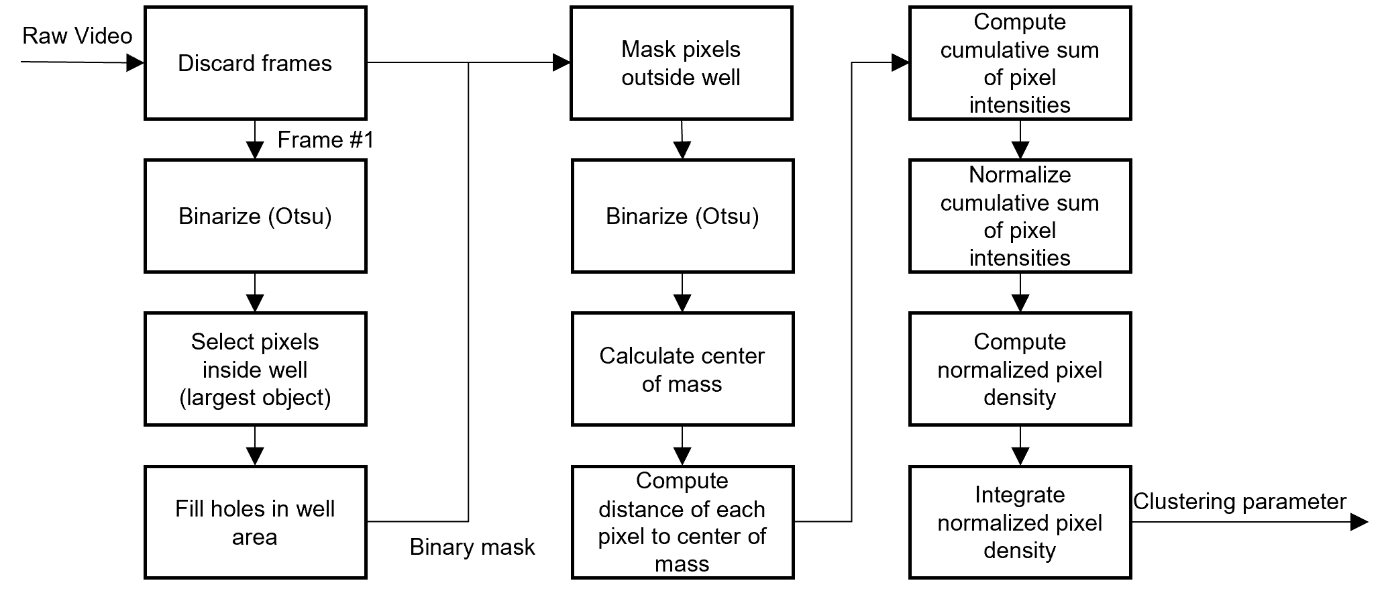


**Figure S7.** Schematic overview of computation steps that were followed to calculate the clustering parameter (*CP*) from a video recording of the emulsion populations.


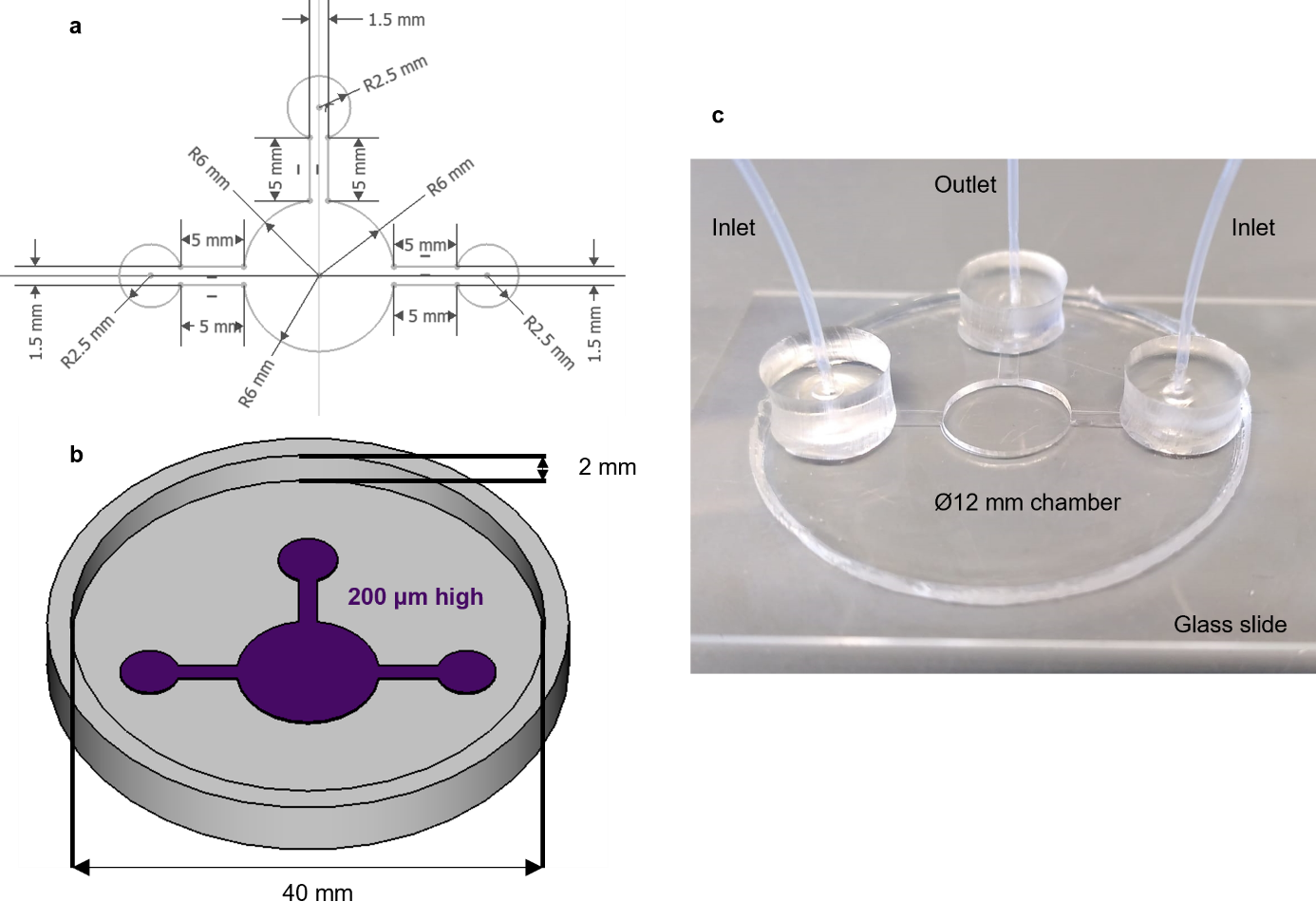


**Figure S8.** Design of PDMS flow chamber. a) Technical drawing with all dimensions for the sample well, the connecting channels and the circular in/outlet chambers. b) 3D drawing of the mold of the PDMS flow chamber. c) Photograph of empty PDMS flow chamber with tubing installed.

**Description of Supporting Videos S1 – S8**

*File Name: Video S1 – Optical microscopy recording corresponding to Figure 2*

Clustering of emulsion droplets upon addition of a base stimulus. Initially, the solution is acidic (pH 2) and the emulsion droplets spread over the surface of the aqueous solution. After addition of a base aliquot (7 μL, 2 m NaOH) at t = 14 s, the solution becomes alkaline (pH 12) and the emulsion droplets move away from the spot where base was added. At the opposite side of the well, the emulsion droplets cluster and propagate towards the centre of the well where they stay.

*File name: Video S2 – Optical microscopy recordings corresponding to Figure S2*

4 different control experiments regarding the emulsion droplet clustering: a) Water is added instead of the base stimulus. Upon addition of water, the droplets do not cluster at all. b) NaCl (7 μL, 2 m) is added instead of the base stimulus. Upon addition of the salt solution, the droplets show slight movement, but not like the clustering after the base stimulus. c) Capstone FS-30 (0.116 wt%) is included in the aqueous solution as a strong surfactant. The emulsion droplets cluster immediately after addition and do not spread at all. d) The pH of the aqueous solution is set to 12.0 prior to application of the emulsion droplets and no further base stimulus is added. The emulsion droplets cluster weakly and spread slowly over time.

*File name: Video S3 – Optical microscopy recordings corresponding to Figure 4a*

Clustering phase diagram for various droplet populations, with different droplet population densities (controlled via total emulsion volume) and loaded with different amounts of 1-heptylamine. The displayed optical microscopy recordings are representative for the data points shown in Figure 4a of the main text.

*File name: Video S4 – Optical microscopy recordings corresponding to Figure 4b*

Optical microscopy recordings for 160 mM 1-heptylamine emulsion droplet swarms applied at a volume of 0.5 μL, 1.5 μL and 3.0 μL on an aqueous FBA solution.

*File name: Video S5 – Optical microscopy recording corresponding to Figure 5*

Dynamic clustering and spreading of the emulsion population (4 µL, 400 mm 1-heptylamine) upon varying base and acid conditions. Base (7 μL, 2 m NaOH) and acid (7 μL, 2 m HCl) are added in multiple cycles to switch the pH from 12 to 2 and form or break down the imine surfactant C_7_-FBA.

*File name: Video S6 – Optical microscopy recordings corresponding to Figure 6*

Spatiotemporal droplet population dynamics in acid-base gradients on an aqueous FBA solution in a PDMS well. In the first and second experiment, both acid (2 m HCl) and base (2 m NaOH) are injected into the well. In the third experiment, we omitted 1-heptylamine from the droplets; in the fourth experiment, we replaced the base inlet for pure water. The emulsion droplets (2 μL) are loaded with 400 mM 1-heptylamine.

*File name: Video S7 – Optical microscopy recordings corresponding to Figure S8*

3 different control experiments regarding the spatiotemporal droplet population dynamics in acid-base gradients in a PDMS well. In the first experiment, the aqueous solution does not contain FBA; base (2 m NaOH) and acid (2 m HCl) are injected into the chamber. In the second experiment, the aqueous solution contains FBA (40 mm, pH 2), while base and water are injected into the chamber. In the third experiment, the aqueous solution contains FBA, and neither acid nor base are fed into the chamber.

*File name: Video S8 – Fluorescence and optical microscopy recordings corresponding to Figure 7*

Optical (brightfield) and fluorescence (385 nm) microscopy of droplet swarms on aqueous solutions with (left) and without (right) 40 mm FBA. Droplet swarms only cluster when FBA is present and subsequently generate a strong fluorescent response due to merging of the two emulsion droplet subpopulations loaded with 1-heptylamine (3 μL, 400 mm) and 7-diethylamino-3-(4'-maleimidylphenyl)-4-methylcoumarin (CPM; 1 μL, 80 μm).
